# Ascorbic acid modulates the structure of the *Pseudomonas aeruginosa* virulence factor pyocyanin and ascorbic acid-furanone-30 combination facilitate biofilm disruption

**DOI:** 10.3389/fmicb.2023.1166607

**Published:** 2023-07-13

**Authors:** Theerthankar Das, Biswanath Das, Brandon Clark Young, Vina Aldilla, Shekh Sabir, Basmah Almohaywi, Mark Willcox, Mike Manefield, Naresh Kumar

**Affiliations:** ^1^Infection Immunity and Inflammation, Charles Perkins Centre, School of Medical Science, The University of Sydney, Sydney, NSW, Australia; ^2^Sydney Institute for Infectious Diseases, School of Medical Science, The University of Sydney, Sydney, NSW, Australia; ^3^Department of Organic Chemistry, Arrhenius Laboratory Stockholm University, Stockholm, Sweden; ^4^School of Chemistry, University of New South Wales (UNSW), Sydney, NSW, Australia; ^5^College of Pharmacy, King Khalid University (KKU), Abha, Saudi Arabia; ^6^School of Optometry and Vision Science, The University of New South Wales, Sydney, NSW, Australia; ^7^School of Chemical Engineering, University of New South Wales (UNSW), Sydney, NSW, Australia

**Keywords:** *Pseudomonas aeruginosa*, biofilm, ascorbic acid, antibiotic resistance, pyocyanin

## Abstract

The production of pyocyanin by *Pseudomonas aeruginosa* increases its virulence, fitness and biofilm formation. Pyocyanin is also a redox molecule and we hypothesize that ascorbic acid being an antioxidant will interact with pyocyanin. The main objective of this study was to investigate the potential interaction of ascorbic acid with pyocyanin, and also to investigate the impact of ascorbic acid in combination with Furanone-30 on quorum sensing and biofilm formation of *P. aeruginosa*. When incubated with ascorbic acid, hyperchromic and hypsochromic shifts in pyocyanin absorbance peaks at 385 nm and 695 nm were observed. In the presence of dehydroascorbic acid and citric acid, these shifts were absent, indicating that the intrinsic antioxidant property of ascorbic acid was probably essential in binding to pyocyanin. NMR spectroscopy showed shifts in ^1^H NMR pyocyanin peaks between 8.2 to 5.8 ppm when incubated in the presence of ascorbic acid. Density Functional Theory (DFT) supported potential interactions between the –CH_2_OH or –OH moieties of ascorbic acid with the –C=O moiety of pyocyanin. The pyocyanin-ascorbic acid complex impaired pyocyanin binding to DNA. Ascorbic acid combined with furanone-30 elevated quorum-sensing inhibition in *P. aeruginosa*, which was directly associated with significantly reduced *P. aeruginosa* virulence, adhesion, aggregation and biofilm formation and enhanced antibiotic-mediated bacterial killing. This study demonstrated that the antioxidant ascorbic acid directly binds to pyocyanin, modulates its structure and results in disruption of biofilm formation and associated tolerance to antibiotics.

## Introduction

*Pseudomonas aeruginosa* is an opportunistic Gram-negative bacterium that can cause life-threatening infections in humans. The World Health Organization (WHO) has placed *P. aeruginosa* in its critical group category due to its infection-causing capability and ability to become multi-antibiotic resistant (WHO, 2017). *P. aeruginosa* is a major pathogen in patients who are immunocompromised including in the early and late stage of HIV infected patients including AIDS patients (Meynard et al., 1999). It is also a major pathogen in cystic fibrosis, bronchitis, burns and surgery-associated wounds patients (Tredget et al., 2004; Ranjan et al., 2010; Fernández-Barat et al., 2021).

It is also a common pathogen responsible for hospital-acquired infections, urinary tract infections (UTIs) and contact-lens-associated keratitis (infection of the cornea), as well as contaminating water and food (Bassetti et al., 2013; CDC, 2023).

One of the major virulence mechanisms of *P. aeruginosa* is its ability to form biofilms. Biofilms consist of cells embedded in an organic matrix, in the case of *P. aeruginosa,* extracellular DNA (eDNA), polysaccharides, proteins, and nucleic acids, are prominent matrix components that facilitate biofilm formation and its structural integrity (Dogsa et al., 2005; Ryder et al., 2007; Flemming and Wingender, 2010; Das et al., 2015). *P. aeruginosa* also secretes various virulence factors such as pyocyanin, hemolysins, elastase, alginate, rhamnolipids, hydrogen cyanide and cyclic dipeptides (Terada et al., 1999; Sato and Frank, 2004; Zulianello et al., 2006; Fothergill et al., 2007; Smith et al., 2013; McCaslin et al., 2015; Chadha et al., 2021; Das, 2021; Kapadia et al., 2022). *P. aeruginosa* also produces ferric (Fe^3+^) iron binding molecules called siderophores (such as pyoverdine) when iron has bioavailability. Bacterial siderophores chelate/scavenge insoluble Fe^3+^ from the environment and solubilize it (*via* reduction) to ferrous (Fe^2+^) iron (Wilson, et al., 2016). Pyoverdine-dependent iron transport affects signalling systems in bacteria which are crucial for biofilm development and also essential for their fitness and growth. *P. aeruginosa* mutants deficient in siderophore production form weak biofilms (Banin et al., 2005; Visca et al., 2007).

Regulation of biofilm formation and virulence factor production can be mediated by a complex cell-to-cell communication system called quorum sensing (QS) (Lee and Zhang, 2015; Fazeli-Nasab et al., 2022). QS involves the synthesis of cellular signalling/autoinducer molecules such as N-acyl homoserine lactones (AHLs) and the *Pseudomonas* quinolone signal (PQS). These molecules bind to receptor molecules that regulate gene transcription (Lee and Zhang, 2015). The PQS system upregulates phenazine-producing operons (genes) *phzA1-G1* and *phzA2-G2,* which produce phenazine-1-carboxylic acid. Phenazine-1-carboxylic acid is converted into pyocyanin by the action of the products of *phzM* and *phzS* (Mavrodi et al., 2001; Lee and Zhang, 2015; Das, 2021).

Pyocyanin (1-hydroxy-5-methyl-phenazine) is an electrochemically (redox) active metabolite and zwitter ion that triggers the formation of reactive oxygen species (ROS) that, in turn, induce oxidative stress and deplete intracellular antioxidant (glutathione) levels in host cells (O’Malley et al., 2004). Pyocyanin production and oxidative stress can increase the production of inflammatory mediators such as interleukin-8, inhibit fibroblast growth and trigger premature cell cycle arrest (senescence) (Denning et al., 1998; O’Malley et al., 2004; Muller et al., 2009; Gonzalez et al., 2016). The overproduction of pyocyanin by *P. aeruginosa* is associated with hyper-virulent strains that produce more severe infections (Fothergill et al., 2007). Pyocyanin is not only a virulence factor but is a crucial factor involved in biofilm formation and biofilm stability (Das et al., 2015). Pyocyanin promotes eDNA release from *P. aeruginosa,* and can bind to DNA and promote electron transfer in *P. aeruginosa* biofilms (Das and Manefield, 2012; Das et al., 2015; Saunders et al., 2020).

Ascorbic acid is a water-soluble antioxidant that scavenges ROS and terminates peroxidative processes (Halliwell and Gutteridge, 1999; Pehlivan, 2017). However, ascorbic acid can also be a pro-oxidant in the presence of metals such as iron and copper triggered by Fenton’s reaction (Buettner, 1993; Bucher and White, 2016). Ascorbic acid has been used to treat bacterial infections such as *Helicobacter pylori*-associated gastritis and peptic ulcers (Mei and Tu, 2018). Ascorbic acid can inhibit the QS mechanism in *P. aeruginosa* and *Vibrio campbellii,* reducing virulence factor production (El-Mofawy et al., 2014; Han et al., 2020).

This study investigated the impact of ascorbic acid directly on pyocyanin and QS in *P. aeruginosa*. A novel discovery is that ascorbic acid interacts directly with pyocyanin and modulates its structure. The complex formation between pyocyanin and ascorbic inhibits pyocyanin binding to DNA. Furthermore, combining ascorbic acid with the QS inhibitor furanone-30 potentiates QS inhibition and results in biofilm disruption and an enhancement of antibiotic-mediated bacterial killing.

## Materials and methods

### Preparation of pyocyanin, ascorbic acid, dehydroascorbic acid and citric acid stock solutions

Pyocyanin (Sigma-Aldrich, Castle Hill, Australia) powder was dissolved at 12% (v/v) in ethanol, and this stock solution was stored in a −30°C freezer. L-ascorbic acid (Sigma-Aldrich) solutions were freshly prepared for each experiment by solubilising in sterile deionised water and adjusting the pH to ~7 with NaOH. Dehydroascorbic acid (oxidised form of ascorbic acid; Sigma-Aldrich) and citric acid (Sigma Aldrich) were prepared by dissolving in MiliQ water and buffered to pH 7 using NaOH.

### Furanone-30 (Fu-30)

Synthetic brominated furanone (Fu 30) which is a potent anti-virulence and quorum sensing inhibitor against *P. aeruginosa* (Manefield et al., 2002; Henzter et al., 2003) was obtained commercially.
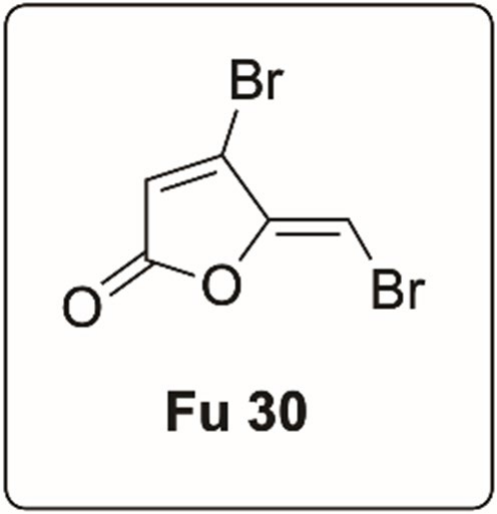


### Pyocyanin-ascorbic acid interaction

Pyocyanin (100 μM) was mixed with ascorbic acid (at both acidic (pH 6–4) and pH 7) in ratios of 1:1 (100 μM:100 μM), 1:20 (100 μM:2000 μM) and 1:100 (100 μM:10000 μM) and incubated in room temperature (25 ± 1°C). The pyocyanin colour was photographed at different time points (0, 24, 48, 72, 96, 120, 144 and 168 h). As a control, pyocyanin is mixed with citric acid [at both acidic (~4) and pH 7] to a ratio of 1:20. Pyocyanin was also mixed with dehydroascorbic acid (pH 7) in ratios of 1:10, 1:20 and 1:100.

To investigate the change in pyocyanin absorbance peaks in the presence of ascorbic acid, citric acid (at both acidic and pH 7) or dehydroascorbic acid, 200 μL of pyocyanin-ascorbic acid mixtures (ratios 1:0, 1:1, 1:5; 1:10 and 1:20), pyocyanin-citric acid mixture (1:20) and pyocyanin-dehydroascorbic acid mixture (1:10, 1:20 and 1:100) were incubated at room temperature (25 ± 1°C) in wells of 96 well plates. The absorbance of the mixtures was recorded between 300–800 nm using a plate reader (Tecan infinite M1000 Pro, ThermoFisher, Scoresby, Australia) at different time points (0, 24, 48, 72, 96, 120 h). The pyocyanin concentration was consistent in all studies (100 μM). The absorbance of ascorbic acid, dehydroascorbic acid and citric acid alone were also recorded at the same time points.

### NMR spectroscopy analysis of pyocyanin-ascorbic acid interaction

To determine the proton NMR spectra of the pyocyanin-ascorbic acid complex, the pyocyanin-ascorbic acid mixture was prepared in sterilised deionised water to a ratio of 1:10 (pyocyanin 100 μM: ascorbic acid 1,000 μM) and incubated for 2 and 72 h at room temperature (25 ± 1°C). After incubation, the samples were loaded in NMR tubes (ThermoFisher), and their ^1^H NMR spectra were recorded using a Bruker Avance 600 MHz spectrometer and applying the solvent (water) suppression technique (see Supplementary material). The chemical shifts (d) in peaks (parts per million; ppm) were recorded.

### Density functional theory analysis of pyocyanin-ascorbic acid interaction

The DFT computational method, based on fundamental laws of quantum mechanics, was used to study the molecular interactions between pyocyanin and ascorbic acid. This work was conducted at Swedish National Infrastructure for Computing (SNIC).

### Chloroform extraction of pyocyanin-ascorbic acid

Pyocyanin-ascorbic acid mixtures were prepared to ratios of 1:20 (pyocyanin 50 μM: ascorbic acid 1,000 μM) and 1:100 (pyocyanin 50 μM: ascorbic acid 10,000 μM). These mixtures were incubated for 2 and 72 h at room temperature (25 ± 1°C), followed by extraction of pyocyanin using the chloroform-HCl assay (Essar et al., 1990; Kapadia et al., 2022). In brief, the pyocyanin-ascorbic acid mixture was mixed with chloroform (Univar., Ingleburn, Australia) to a dilution factor of 300 μL of chloroform for every 1,000 μL of the mixture in 15 mL falcon tubes and vigorously shaken manually and vortexed immediately for 5 s followed by centrifugation at 4500 × g for 10 min at 4°C. Chloroform separates residual pyocyanin in the mixture into a blue layer. The blue layer was carefully pipetted out into a new tube and then treated with 0.2 M hydrochloric acid (HCl) at a ratio of 500 μL of 0.2 M HCl for every 1,000 μL of blue solution. The HCl-blue pyocyanin mixture was again shaken manually, then immediately vortexed for 5 s and centrifuged for further 10 min (4,500 × g; 4°C). The final acidified pyocyanin appeared as a top pink layer, and 200 μL of that pink layer was added to the wells of 96 well plates, and its absorbance was recorded at 520 nm using a plate reader. The absorbance given by pure pyocyanin was used taken as 100%, and the absorbance of the pyocyanin-ascorbic acid mixture was assessed with respect to pure pyocyanin.

### The study of the interaction of pyocyanin and phenazine to DNA using circular dichroism

A Chirascan Circular Dichroism spectrophotometer (Applied Photophysics, Applied Photophysics Limited, Surrey, United Kingdom) was used to investigate the interaction between pyocyanin or phenazine and DNA. Mixtures of calf thymus double-stranded (ds) DNA (Sigma Aldrich, Australia) at 150 ng/μL with varying pyocyanin or phenazine (0, 25, 50 and 100 μM) concentrations in Milli-Q water were incubated at room temperature (25 ± 1°C) for 15 min. Following incubation, the absorbance of the mixture was scanned from 200–320 nm (at a scan rate of 0.5 s/nm) in a 1 mm path-length quartz cuvette. Shifts in the DNA absorbance peaks due to pyocyanin/phenazine binding was recorded and plotted as CD-mdeg vs. wavelength-nm.

### Impact of ascorbic acid on DNA

The effect of ascorbic acid on ds DNA was investigated using fluorometry. dsDNA (200 ng/μL) was incubated for 2 and 72 h at room temperature (25 ± 1°C), in either presence or absence of ascorbic acid (50 and 1,000 μM) at both intrinsic and neutral pH. After 2 and 72 h, the dsDNA concentration was quantified using a fluorescent dye assay (dsDNA BR; Qubit, Invitrogen) and monitored with a Qubit 3.0 Fluorometer (Invitrogen, Life Technologies, Carlsbad, CA, United States). As controls, pyocyanin (50 μM), pyocyanin-ascorbic acid mixture to a ratio of 1:1 and 1:20 and DNase I-50 units (DNase I recombinant, RNase-free from Roche, Merck) were also incubated with ds DNA, and any depletion in dsDNA concentration was recorded at 2 and 72 h.

### Ethidium bromide displacement method to analyse the impact of ascorbic acid on DNA-pyocyanin interaction

The ability of ascorbic acid to hinder pyocyanin binding to ds DNA was assessed using the ethidium bromide (EtBr)-DNA displacement technique (Das et al., 2015). Double-stranded DNA was mixed with ethidium bromide (4 mM) in SHE buffer (2 mM HEPES,10 mM EDTA and 9.4 mM NaCl in Milli-Q water, adjusted to pH 7 with NaOH), and its fluorescence emission at 615 nm (excitation at 480 nm) was quantified at room temperature (25 ± 1°C) in a 1 mL quartz cuvette using a Varian Cary Eclipse Fluorescence Spectrophotometer, Agilent, United States. Where indicated, either deionised water or pyocyanin (100 mM) or pyocyanin-ascorbic acid mixture (pH 7, incubated for 2 h at ratios 1:2 and 1:20) was added to the EtBr-DNA complex, and a change in fluorescence signal was recorded. The change in fluorescence signal with respect to control (EtBr-DNA) determines the pyocyanin or pyocyanin-ascorbic acid mixture’s ability to displace EtBr from DNA and binding of pyocyanin to DNA.

### *Pseudomonas aeruginosa* isolates and growth conditions

The *P. aeruginosa* isolates used in this study were *P. aeruginosa* MH602 *las*B::*gfp*(ASV) for LasR analysis, PAO1 rhlA::gfp for RhlR analysis, PAO1 *pqs*A::*gfp* for PqsR analysis, and the clinical isolates DFU-53 (diabetic leg wound ulcer, Liverpool Hospital, NSW, Australia), wound-364,077 (head wound), and the urinary tract isolates UTI-15 and UTI-62 (Royal Prince Alfred Hospital, NSW, Australia). These isolates were stored in a −80°C freezer in 25% DMSO. For this study, 25 μL of the stock was directly plated onto Tryptone soy (TS, Oxoid, Australia) agar plates and incubated for 24 h at 37°C. Single colonies were inoculated into 5 mL of full-strength Mueller Hinton Broth (MHB) and incubated for 24 h at 37°C with shaking (100 rpm). For the isolates, MH602 *las*B::*gfp*(ASV), PAO1 *rhl*A*::gfp* and PAO1 *pqs*A::*gfp* were grown in the presence of 30 μg/mL gentamicin.

### Effect of ascorbic acid and Fu-30 on *Pseudomonas aeruginosa* QS and growth

After growth in MHB, *P. aeruginosa* isolates MH602 *las*B::*gfp*(ASV), PAO1 *rhl*A*::gfp* and PAO1 *pqs*A::*gfp* were diluted to a ratio of 1:4 in M9 minimal salts media (Sigma-Aldrich, Sydney, NSW, Australia). Note that M9 media was chosen due to its low autofluorescence and the ratio of 1:4 of MHB:M9 used in this study was found to be ideal for *P. aeruginosa* growth whilst at the same time allowing for the measurement of GFP production with minimal autofluorescence interference from MHB to a total volume of 5 mL in a six-well plate (Corning Corp., Corning, NY, United States) to an optical density (OD_600 nm_) 0.1 ± 0.02, either in the presence or absence of ascorbic acid (10, 20, 30, 40, 50 and 70 mM), Fu-30 (10, 20, 30, 40, 50, and 100 μM) or combinations of ascorbic acid-Fu-30 in ratios of 10 mM + 10 μM, 10 mM + 20 μM, 20 mM + 10 μM and 20 mM + 20 μM. For untreated control and to test the impact of solvent, PBS and DMSO (equivalent to respective Fu-30 concentrations) were used in place of the Fu-30. Finally, 200 μL of the diluted bacterial media were dispensed into the 96-well plates (Corning Corp., Corning, NY, United States) and incubated at 37°C with orbital shaking at 100 rpm. Production of GFP *via* the receptor proteins LasR, RhlR and PqsR and bacterial growth was monitored every hour for 8 h and then after 24 h by measuring fluorescence at Ex_488 nm_ and Em_515 nm_ for LasR, RhlR and PqsR and OD_600 nm_ for bacterial growth using a plate reader. To quantify LasR, RhlR and PqsR expression, the GFP fluorescence values measured at every time point were divided by their respective OD_600 nm_ absorbance. A graph was plotted as GFP/OD vs. time in hours.

### Pyocyanin extraction from *Pseudomonas aeruginosa* grown in ascorbic acid-Fu 30 combinations

*P. aeruginosa* isolates MH602, PAO1, DFU-53, wound, UTI-62, and UTI-15 were grown in 20% MHB for 24 h in the presence or absence of ascorbic acid. After 24 h, the cultures were centrifuged at 4500 × g for 10 min at 25°C to separate the bacterial cell pellet from the supernatant. The amount of pyocyanin in the supernatant was quantified using the chloroform-HCl assay.

### The effect of ascorbic acid-Fu-30 on *Pseudomonas aeruginosa* hemolysin activity

*P. aeruginosa* isolates were grown in the presence or absence of ascorbic acid (20 mM) + Fu-30 (20 μM) for 24 h. Cell-free supernatants were collected by centrifugation at 4500 × g for 10 min at 25°C followed by filtering through a 0.22 μm Millex-GP syringe filter (Millipore, Merck, Australia). In parallel, washed rabbit blood was prepared by mixing 2 mL of rabbit blood (Equicell, Australia) with 13 mL of PBS in a 50 mL falcon tube, followed by centrifugation (4,500 × g for 5 min at 25°C). After centrifugation the supernatant was discarded and the washing procedure with PBS by centrifugation was repeated three times. Finally, the washed rabbit blood pellet was resuspended in 5 mL of PBS for further hemolysin assay. The cell-free supernatants were added to the 100 μL of washed rabbit blood at ratios of 0.25:1, 0.5:1 and 1:1. These were then incubated for 60 min at 37°C with shaking (100 rpm). After centrifugation for 10 min at 4500 × g at 25°C, 100 μL of the supernatant was transferred into 96-well plates, and hemolysis of the red blood cells was quantified by measuring absorbance at 520 nm.

### Effect of ascorbic acid-Fu-30 on *Pseudomonas aeruginosa* adhesion and biofilm formation

*Pseudomonas aeruginosa* isolates were inoculated to an optical density (OD_600 nm_) = 0.1 ± 0.02 and allowed to grown in 20% MHB in the presence or absence of ascorbic acid (20 mM) + Fu-30 (20 μM) for 2, 8 and 24 h at 37°C with shaking (100 rpm) in 6-well plates. At the respective time points, the supernatant was removed, and the plates were washed three times with PBS to remove any loosely adhered bacteria. Images of the bacteria in the well were captured using a light microscope (Zeiss AxioScope.A1 FL, LED, Jena, Germany) and the total area of bacterial adhesion were quantified using Fiji Image J software. After imaging, crystal violet (0.05% w/v) was added to the wells and incubated overnight at 37°C and 100 rpm. The crystal violet dye was then removed from the wells, which were washed wells three times with PBS to remove excess crystal violet. The wells were dried at 37°C for 30 min followed by adding 1,000 μL of 80% ethanol (to dissolve crystal violet) and incubated further for 60 min at 37°C with shaking (100 rpm). The dissolved crystal violet was then transferred into a new 96-well plate, and absorbance was recorded at OD_550 nm_. The biofilm formed in the absence of ascorbic acid and Fu-30 was used as a control (100% biomass), and variations in biofilm biomass with ascorbic acid (20 mM) + Fu-30 (20 μM) combination-treated biofilms were assessed with respect to the control.

### Effect of ascorbic acid + Fu-30 on *Pseudomonas aeruginosa* colony forming ability

*Pseudomonas aeruginosa* isolates were grown (as described above) in the presence or absence of ascorbic acid (20 mM) + Fu-30 (20 μM) for 2, 8 and 24 h, followed by washing with PBS. Adhered bacteria were removed from the incubation vessels by scrapping a sterile surgical knife 30 times over the surface in 5 mL of PBS. The bacterial suspension was serially diluted in PBS and incubated on TS agar at 37°C for 24 h. The number of CFU/mL was calculated by counting the individual colonies on the agar plates to determine the number of surface-adhered bacteria grown in the presence or absence of ascorbic acid-Fu 30.

### The impact of tobramycin on pre-established biofilms

*Pseudomonas aeruginosa* clinical isolates DFU-53 and UTI-62 biofilms were grown in 20% MHB in 6-well plates in the presence or absence of ascorbic acid (20 mM) + Fu-30 (20 μM) for 24 h, followed by washing three times in PBS. The attached biofilm was then treated with tobramycin (1, 5 and 10 × MIC, MIC values of *P. aeruginosa* DFU-53 and UTI-62 were mentioned in Supplementary data) in PBS and incubated for 4 h at 37°C and 100 rpm. After 4 h, the tobramycin-treated biofilms were washed twice with PBS, followed by either staining with crystal violet for biofilm biomass quantification or scrapping off adhered cells and incubating on TSB agar (as outlined above).

### Harvesting human foreskin fibroblast cells (HFF-1)

HFF-1 (passage #15–17) was cultured in DMEM medium supplemented with fetal bovine serum (12% v/v), penicillin (100 IU/mL) and streptomycin (100 μg/mL) at 37°C in a 5% (v/v) CO_2_ and harvested at 95% confluence using 0.12% v/v trypsin-EDTA. Cells were collected after quenching Trypsin 1:1 v/v with media and transferred to 50 mL falcon tubes, followed by centrifugation (6 min, 4,500 × g, 20°C). The supernatant was aspirated, and the HFF-1 cell pellet was suspended in supplemented DMEM media for further experiments. HFF-1 cells were plated into six-well plates to a density of 10^5^ cells/mL and allowed to incubate at 37°C in a 5% (v/v) CO_2_ under static conditions, with the fresh supplemented DMEM growth medium being added every alternative day until a confluence of 95%–100% of cells (verified using a light microscope) were achieved.

### Analysis of the effect of ascorbic acid-Fu-30 in human foreskin fibroblast cells (HFF-1)

HFF-1 cytotoxicity was evaluated using the British Standard (ISO 10993-5:2009, 2009)—direct contact assay. At 95%–100% confluence, the cells were supplemented with 1 mL of the growth medium, followed by placement of a freshly made antibiotic filter disc (6 mm, Whatman GE Healthcare, Sydney, Australia) impregnated (incubation at ambient temperature for 2 h) with 50 μL of the combination in the centre of the wells. The filter disc was prepared by adding 50 μL of test compounds directly onto 6 mm antibiotic filter paper discs and incubating them at room temperature (25 ± 1°C) for 2 h to allow the discs to absorb test compounds. The plates were then incubated for 24 h at 37°C in a 5% (v/v) CO_2_. After 24 h, the filter disc and the cell growth media were removed and replaced with 1 mL sterile PBS. The cell morphology of the HFF-1 cells and their confluence were assessed by light microscopy. For the controls, a filter disc containing 50 μL 0.9% w/v NaCl and another containing 50 μL 100% DMSO (a positive control that causes cell death) were used. Based on ISO 10993-5:2009 (2009), test compounds were considered non-cytotoxic if the viability of cells remained above 80% (grade 0) and mild if cell lysis was not more than 50% (grade 2). The viability of HFF-1 cells was also quantified using a resazurin assay. To measure HFF-1 metabolic viability, HFF-1 cells were washed once by simply adding 1 mL sterile PBS to the HFF-1 cells and allow it incubate in the room temperature for 1 min, followed by pipetting out the PBS, and immediately replacing it with 1 mL of 0.05% w/v resazurin solution (pre-prepared by dissolving resazurin in sterile PBS and stored in fridge at 4°C and used as required) for 24 h staining by incubating at 37°C in a 5% (v/v) CO_2_. After 24 h incubation, the fluorescence intensity of the HFF-1 was measured using a Tecan plate reader at Ex _544 nm_ and Em_590 nm_.

### Statistical analysis of data

All experiments were conducted in triplicate. Statistical evaluation of all results was performed using an unpaired *t-*test in GraphPad prism.^1^ The results were considered statistically significant and indicated with “* (asterisk)” if *p* < 0.05.

## Results

### Effect of ascorbic acid on pyocyanin absorption spectra

**Figure 1:**
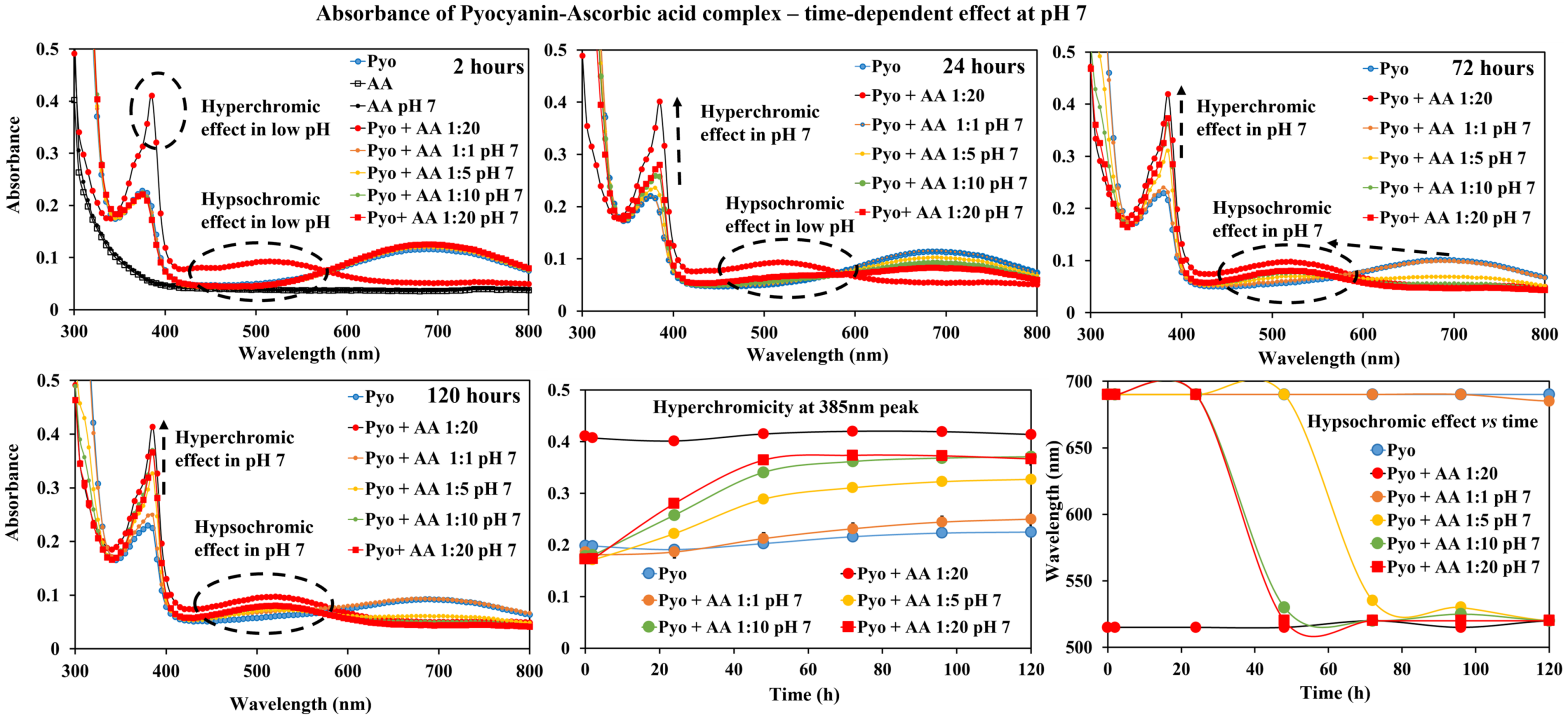
Spectrophotometer analysis of ascorbic acid (AA) impact on pyocyanin (Pyo) absorbance. Ascorbic acid (acidic pH) showed immediate hyperchromic and hypsochromic shifts of pyocyanin absorbance peak at 385 nm and 695 nm, respectively. In the presence of neutralised ascorbic acid, the change was gradual and was more prominent at later hours. The bottom centre and right-end graphs show a summary of the hyperchromic shift and hypsochromic shift of pyocyanin absorbance peak at 385 and 695 nm in the presence of ascorbic acid over time.

Figure 1 shows the associated changes in pyocyanin absorbance at different time points (2, 24, 72 and 120 h). Pyocyanin has absorbance peaks at 385 nm and 690 nm, whilst ascorbic acid has no such features. When mixed with ascorbic acid without buffer, the peak at 385 nm intensified (reached up to absorbance value 0.4) and the broad peak of pyocyanin at 690 nm rapidly shifted to 510 nm immediately (as seen in 2 h time point) and remained in the intensified and shifted position throughout the study (recorded over 120 h).

In buffered solution (neutral pH), ascorbic acid addition resulted in a more gradual change in intensity of the pyocyanin peak at 385 nm and shift of the broad peak. For example, at 2 h time point no change in pyocyanin peaks at both 385 and 690 nm, whereas at 24 h time point hyperchromic effect (i.e., increase in absorbance value at 395 nm peak) was observed and at 72 h and 120 h time points both hyperchromic effect and hypsochromic shift in pyocyanin peaks were evident. To note the impact of ascorbic acid (neutral pH/buffered) on pyocyanin is concentration dependent.

### NMR of pyocyanin-ascorbic acid complex

**Figure 2:**
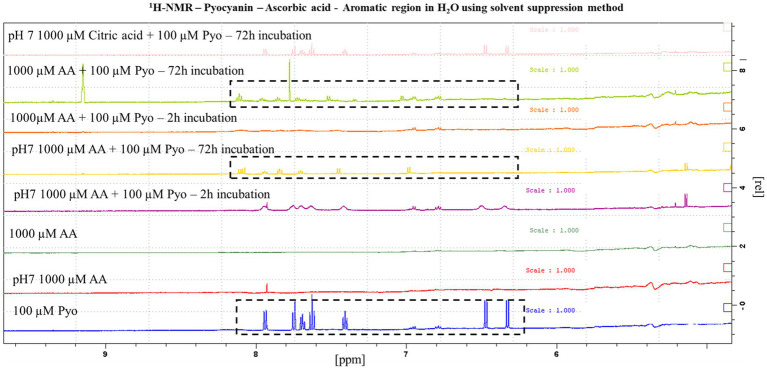
^1^H-NMR analysis of the impact of ascorbic acid (AA) on pyocyanin in the pyocyanin aromatic region. The aromatic part of pyocyanin showed precise modulation in its characteristic NMR signals upon forming a complex with ascorbic acid at both acidic and physiological pH. Main y after 72 h of formation of new peaks at 8.1 ppm and disappearance of pyocyanin peaks around 6.2 and 6.4 ppm refer towards the construction of new species or a pyocyanin-ascorbic acid adduct.

NMR spectroscopy shows clear changes in the pyocyanin (at pH 7, 100 μM) structure upon adding ascorbic acid (1,000 μM). After 2 h, all the characteristic pyocyanin NMR signals were broadened, minor peaks around 6.6–7.0 ppm (possible impurity in pyocyanin) remained intact, and new peaks around 5.2 ppm appeared (Figure 2). The complete disappearance of the pyocyanin peaks (around 6.2 and 6.4 ppm) and the appearance of new sets of NMR signals were observed after 72 h (at pH 7) as the result of the formation of a new species or a pyocyanin-ascorbic acid adduct. When the pH was not controlled, a similar change in the NMR signals was seen after 2 h, but after 72 h two additional signals at around 9.2 and 7.7 ppm were seen. Further detailed spectroscopic investigations are needed to determine the exact identity of these new signals. Nevertheless, the structural change of pyocyanin in the presence of ascorbic acid (Figure 2) was confirmed by these NMR experiments. No such changes were observed when ascorbic acid was replaced by citric acid (at pH 7), even after 72 h of incubation under similar conditions.

### Density functional theory prediction on pyocyanin-ascorbic acid interactions

**Figure 3:**
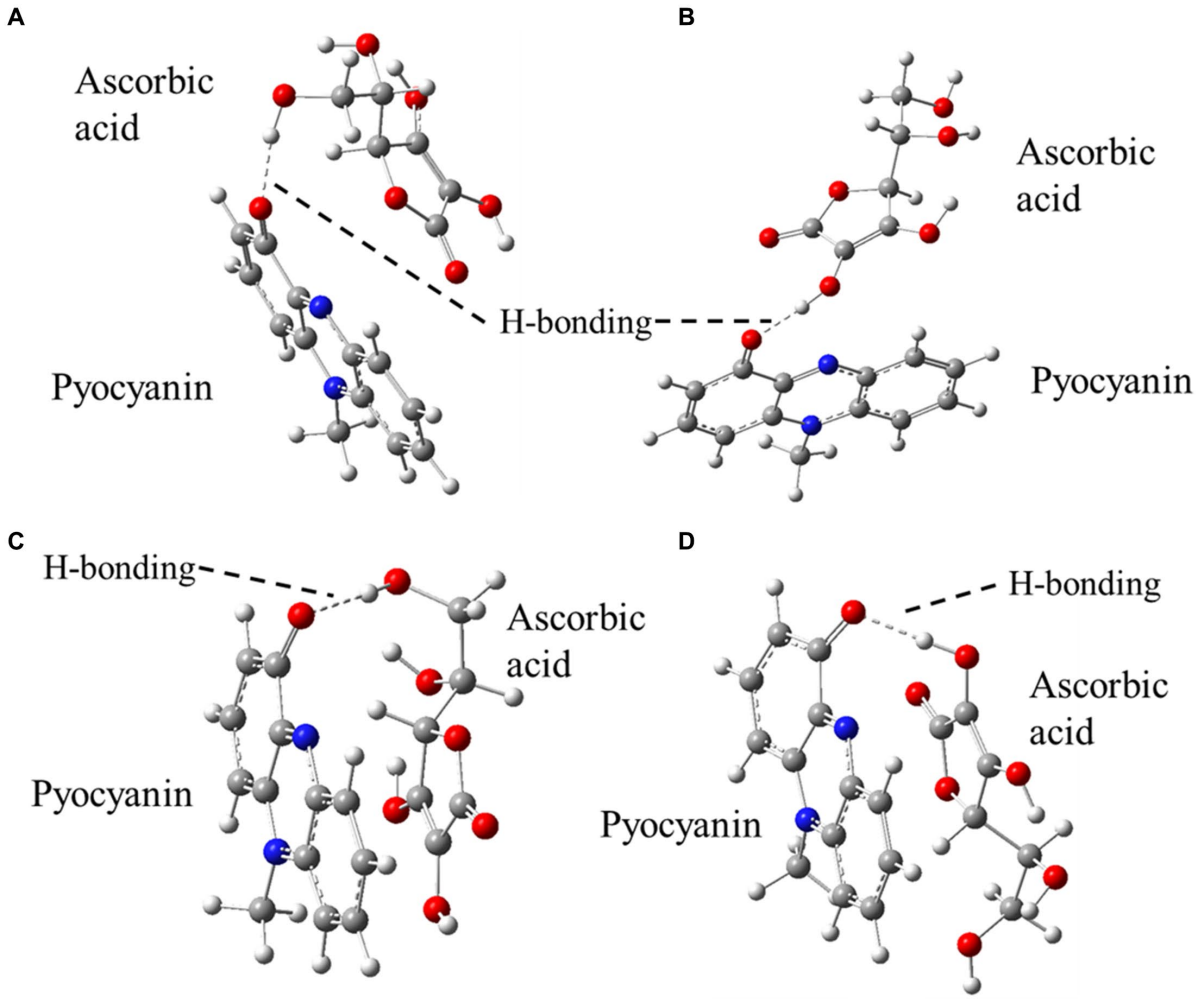
DFT optimised structures of the possible H-bonding interactions between pyocyanin and ascorbic acid. A-D represents the possibility of H-bonding between pyocyanin and ascorbic acid.

Density functional theory (DFT) predicted two feasible H-bonding interactions between (i) the –CH_2_OH unit of ascorbic acid with –C=O of pyocyanin and (ii) the most acidic –OH unit of ascorbic acid with –C=O of pyocyanin (Figure 3). Both these structures were optimised (unrestricted) by DFT using the B3LYP (Becke, 1993), function as implemented in the Gaussian 09 program and 6-31G(d,p) basis set for all elements, applying the aqueous solvation (SMD) model (Kromann et al., 2018). According to these calculations, both the orientations are of almost similar energies, favouring slightly (by 1.27 kJ/mol) the H-bond (with 1.73 Å bond distance) from –CH_2_OH of ascorbic acid (Figure 3A) over the other (with 1.63 Å bond distance) (Figure 3B). Another function, B97D3 (Antony and Grimme, 2006), which has dispersion correction and is reported to be more accurate for pi–pi stacking interactions (unrestricted, including SMD model for aqueous solvation), identified that the H-bond (with 1.88 Å bond distance) from –CH_2_OH of ascorbic acid (Figure 3C) was more favourable (by 13.01 kJ/mol) over the other (with 1.71 Å bond distance) (Figure 3D). The full calculations can be found in Supplementary Figure S4.

### Effect of ascorbic acid on pyocyanin yield

**Figure 4:**
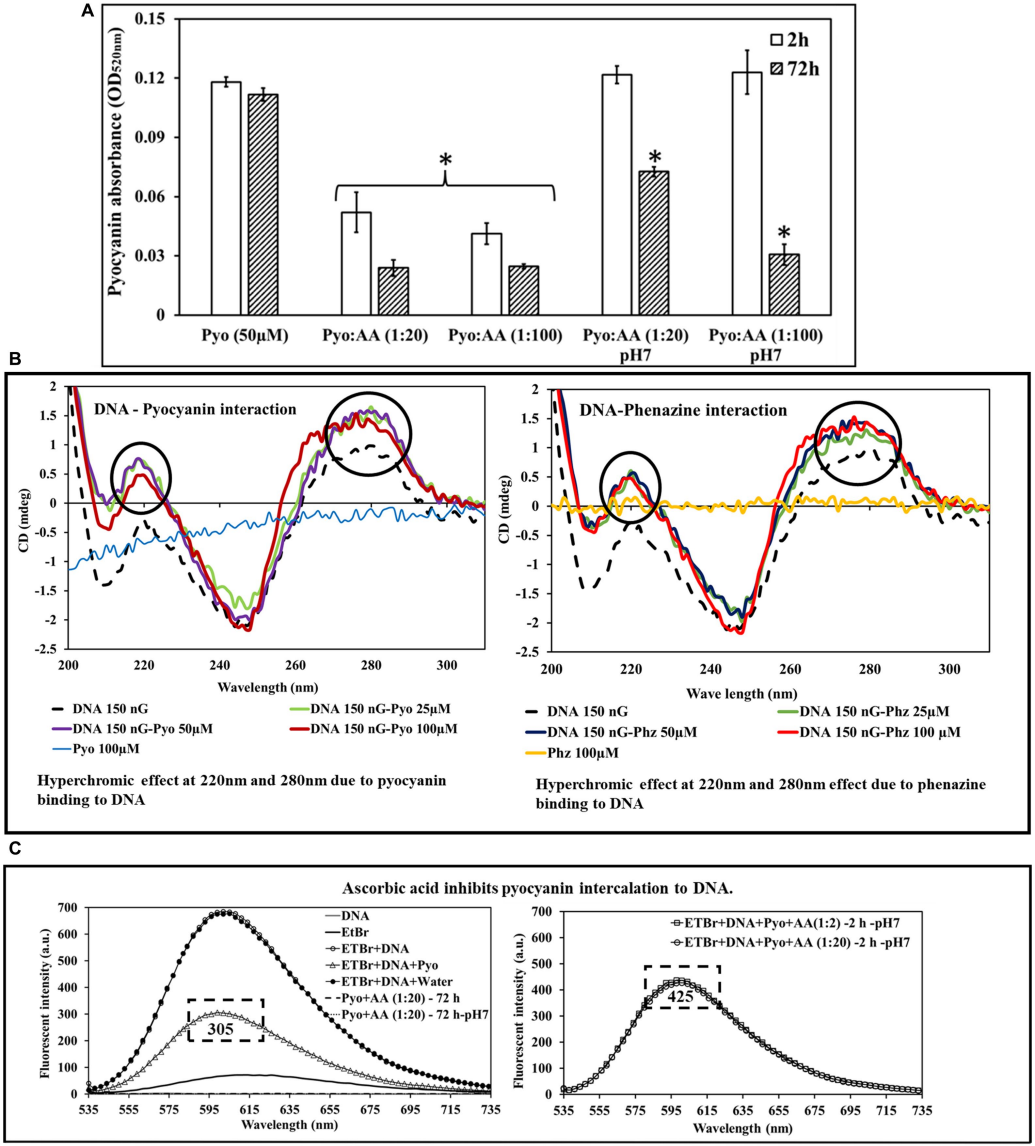
Pyocyanin incubated with ascorbic acid (AA) for 2 and 72 h in the presence of acidic or neutral pH showed a drastic reduction in pyocyanin recovery. **(A)** The impact is higher and faster with acidic ascorbic acid than with its buffered version. ^*^Indicates the differences are statistically significant (*p* < 0.05) compared to pyocyanin 50 μM. All experiments were conducted with *n* = 3 replicates. **(B)** Circular dichroism shows pyocyanin and phenazine intercalate with DNA, as evident by the hyperchromic effect observed at pyocyanin peaks at 220 nm and 280 nm. **(C)** Fluorescent spectroscopy analysis showing ascorbic acid inhibits pyocyanin binding to DNA. EtBr displacement method demonstrates pyocyanin displaces EtBr and binds with DNA (empty circle) and fluorescent intensity down to 305 (left panel). In the presence of AA, the Pyo-AA complex hinders pyocyanin binding to DNA with a fluorescent value raised to 425 (right panel).

The amount of pyocyanin, when mixed with ascorbic acid and incubated for 2 or 72 h, was significantly reduced (Figure 4A). This was less pronounced when the aqueous mixture was buffered to neutral pH, but was time and concentration-dependent. These results suggest that ascorbic acid reacts with pyocyanin in neutral aqueous environments.

### Ascorbic acid inhibits pyocyanin binding to DNA

Circular dichroism spectra showed a shift in the characteristic dsDNA peaks in the presence of pyocyanin and phenazine consistent with dsDNA binding (Figure 4B). The most prominent shifts in mdeg were observed at around 209, 220 nm and 280 nm. The data suggest pyocyanin and phenazine create local modulations in the DNA structure (Figure 4B). Fluorescence spectroscopy (Figure 4C) demonstrated that the fluorescence maximum for EtBr bound to dsDNA decreased in intensity in the presence of pyocyanin, consistent with pyocyanin displacing intercalated EtBr. When ascorbic acid was present, pyocyanin did not reduce fluorescence intensity, consistent with ascorbic acid interfering with pyocyanin intercalation through complexation. Note that in an unbuffered solution, ascorbic acid can cause DNA fragmentation (Erdem et al., 1994) (Supplementary Figure S5D); hence all experiments were undertaken at neutral pH.

### The combination of ascorbic acid and Fu-30 inhibited the function of QS regulatory receptors

**Figure 5:**
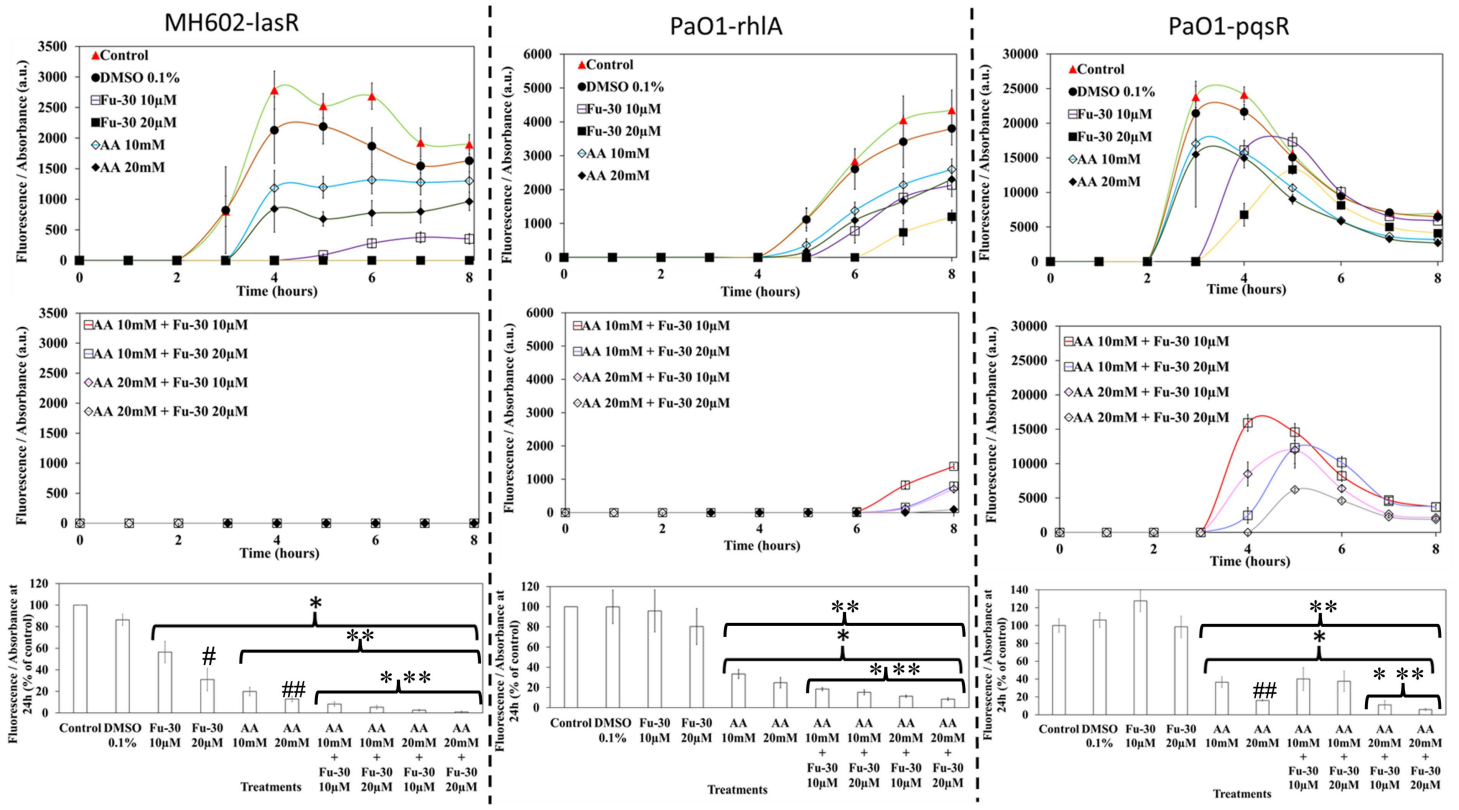
Impact of ascorbic acid (AA) and furanone-30 (Fu-30) and combination of both on *P. aeruginosa* quorum sensing. MH602 was used to study the effects of the lasR system (left hand side), whereas PAO1 was used to study rhlA (centre) and pqsR (right hand side). Fu-30 showed a decline in the production of GFP up to 8 h and the addition of AA alone showed considerable declines in GFP production at all time points. The combination of AA with Fu-30 showed the most decrease in the activity of all three QS systems, especially at AA 20 mM + Fu-30 20 μM. All experiments were conducted in triplicate. ^*^Indicates *p* < 0.05 in comparison to the control, ^**^indicates *p* < 0.05 in comparison to the Fu-30 alone and ^***^p < 0.05 in comparison to the AA alone, ^#^indicates *p* < 0.05 comparison between increasing concentration of Fu-30 alone and ^##^*p* < 0.05 comparison between increasing concentration of AA alone.

The individual compounds ascorbic acid and known QS inhibitor Fu-30, as well as a combination of both, inhibited QS response regulators LasR, RhlA and PqsR mediated GFP production (Figure 5). At times up to 8 h, Fu-30 was more effective than ascorbic acid in inhibiting GFP production. However, by 24 h ascorbic acid became more effective than Fu-30. Interestingly, the combination of ascorbic acid and Fu-30, especially at the highest concentration (ascorbic acid 20 mM + Fu-30 20 μM), showed the greatest reduction in GFP production in comparison to Fu-30 and ascorbic acid treatment alone for all three QS receptors at every time point.

### The combination of ascorbic acid and Fu-30 inhibited pyocyanin production

**Figure 6:**
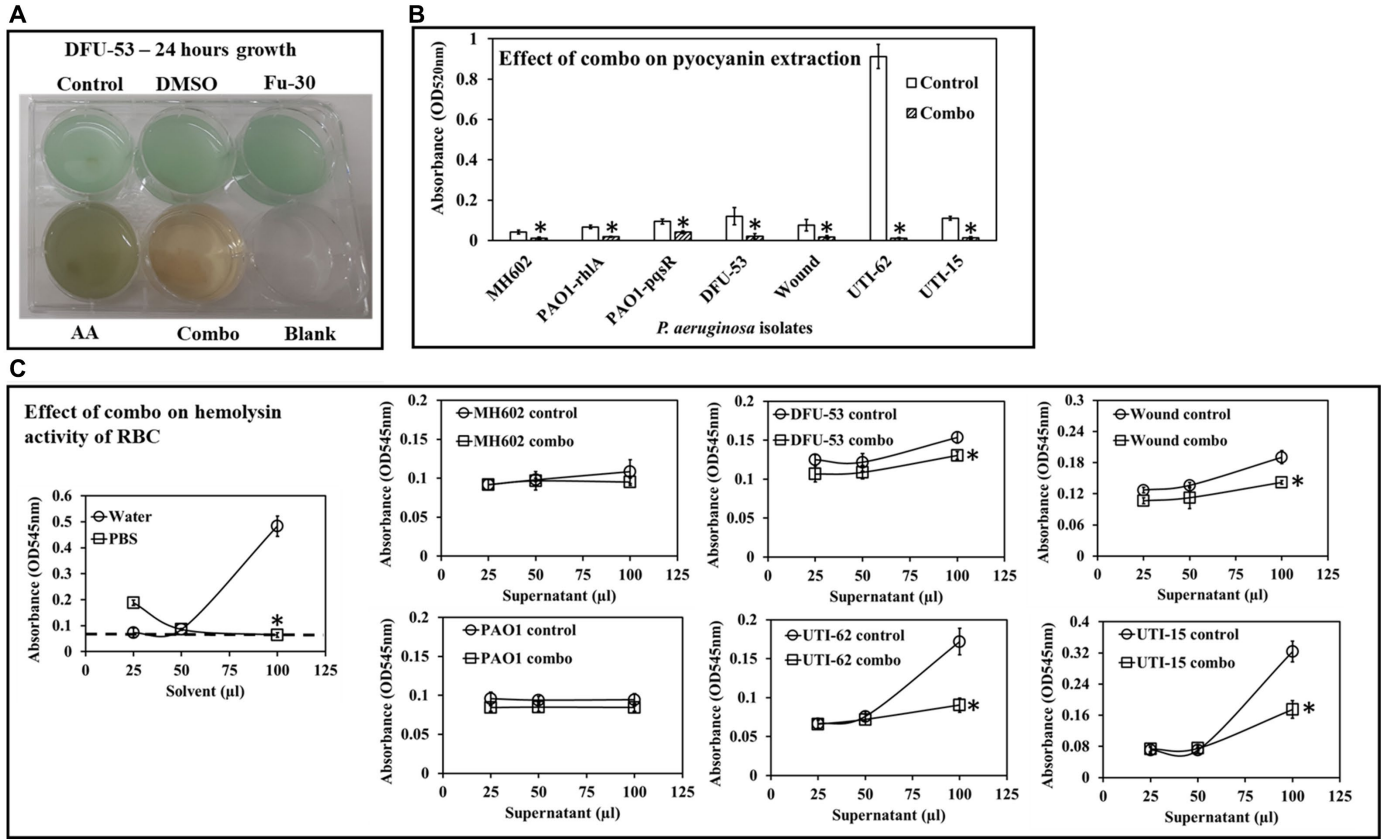
Impact of the combo [combination of AA (20 mM) and Fu-30 (20 μM)] on *P. aeruginosa* virulence factor pyocyanin and hemolysin. **(A)** Only combo shows the disappearance of greenish colour (an indicator of pyocyanin production) in bacterial culture. **(B)** Combo significantly decreased pyocyanin production in all *P. aeruginosa* strains/isolates. **(C)** Combo also significantly decreased hemolysin activity (at the highest volume of supernatant used at 100 μL) in all clinical isolates. Experiments were conducted in triplicates. ^*^Indicates *p* < 0.05.

Analysis of Pyocyanin production by *P. aeruginosa* isolates grown for 24 h in the presence or absence of the combination of ascorbic acid and Fu-30 (Figure 6A). There was a significant decrease in pyocyanin yield in both clinical and laboratory isolates when grown in the presence of the combination. When untreated, the *P. aeruginosa* strains’ pyocyanin production as indicated by optical density ranged from (OD_520 nm_) 0.04 to 0.9, with UTI-62 having the highest pyocyanin production. Whereas when treated with the combination of ascorbic acid and Fu-30, all strains had a significant decrease in pyocyanin production (OD_520 nm_) of 0.018 (Figure 6B).

### The combination treatment inhibited hemolysin activity

Figure 6C shows the laboratory strains MH602 and PAO1 did not produce hemolysin since there absorbance (OD545 _nm_) is approximately 0.1 (similar to the base line) whereas, the clinical isolates produced hemolysin. When grown in the presence of the combination of ascorbic acid and Fu-30, the hemolytic activity of the clinical isolates decreased, with the decrease being more prominent among UTI isolates and at a 1:1 volume ratio (i.e., 100 μL blood: 100 μL *P. aeruginosa* cell-free supernatant). Whereas, at lower ratio of *P. aeruginosa* cell free supernatant to rabbit blood did not show any hemolytic activity.

### The combination treatment reduced initial adhesion

**Figure 7:**
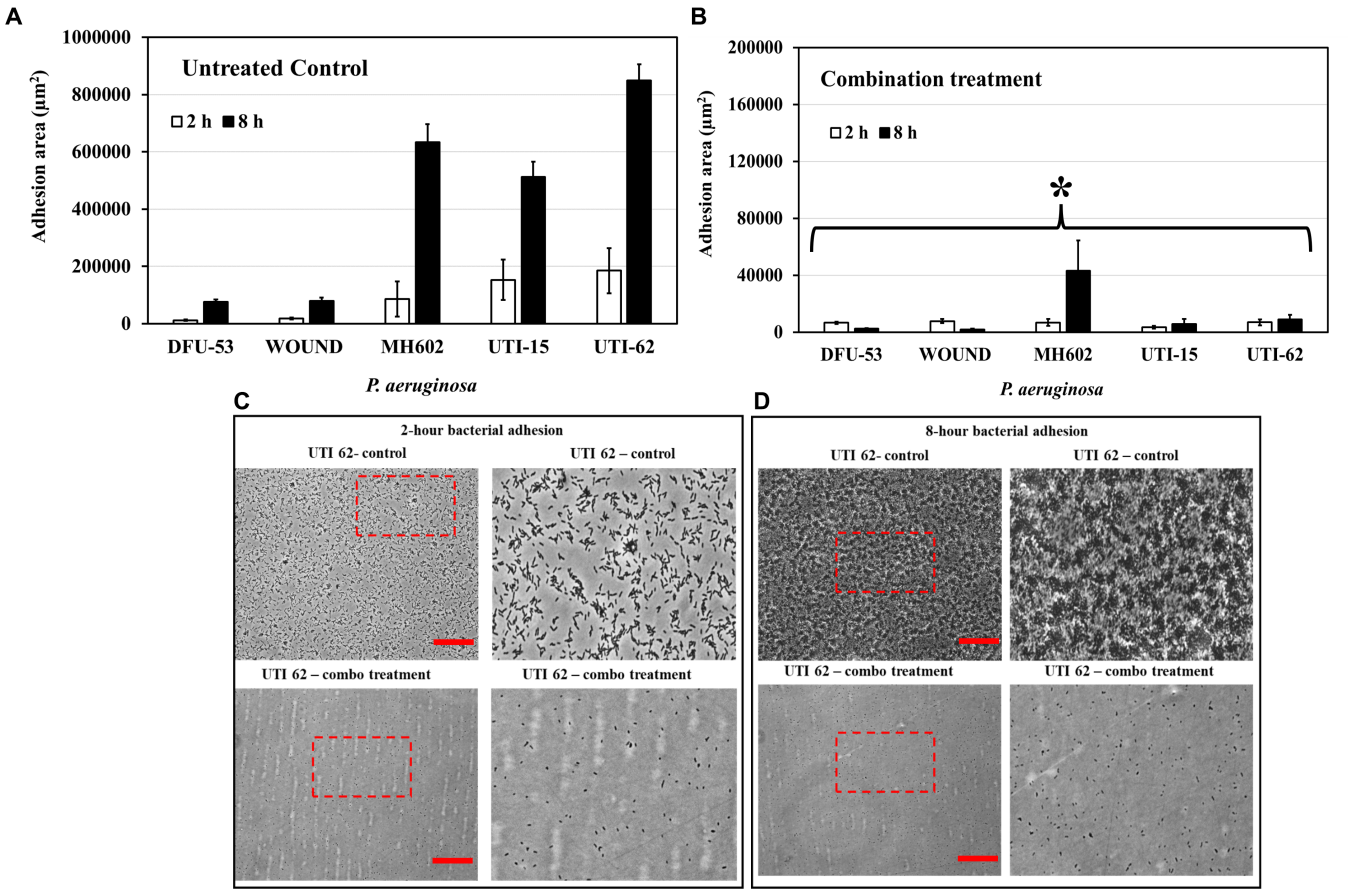
Impact of combination treatment on *P. aeruginosa* adhesion. **(A)** The adhesion area of *P. aeruginosa* increased significantly for all isolates from 2 h to 8 h. **(B)** In the presence of a combo, the bacteria’s total adhesion area stagnated, and the value remained the same for 2 and 8 h time points. **(C,D)** The microscopic image represents an example of *P. aeruginosa* isolate (UTI-62) adhesion at 2 and 8 h time points. Scale bar = 50 μm. ^*^Indicates the differences are statistically significant (*p* < 0.05) compared to the untreated control. All experiments were conducted in *n* = 3 biological replicates.

When grown in the presence of the combination of ascorbic acid and Fu-30 there was a significantly reduced adherence of bacteria especially after 8 h incubation for all isolates (Figures 7A,B). Figures 7C,D shows representative images of UTI-62 after 2 and 8 h incubation.

### The combination treatment reduced *Pseudomonas aeruginosa* biofilm-biomass

**Figure 8:**
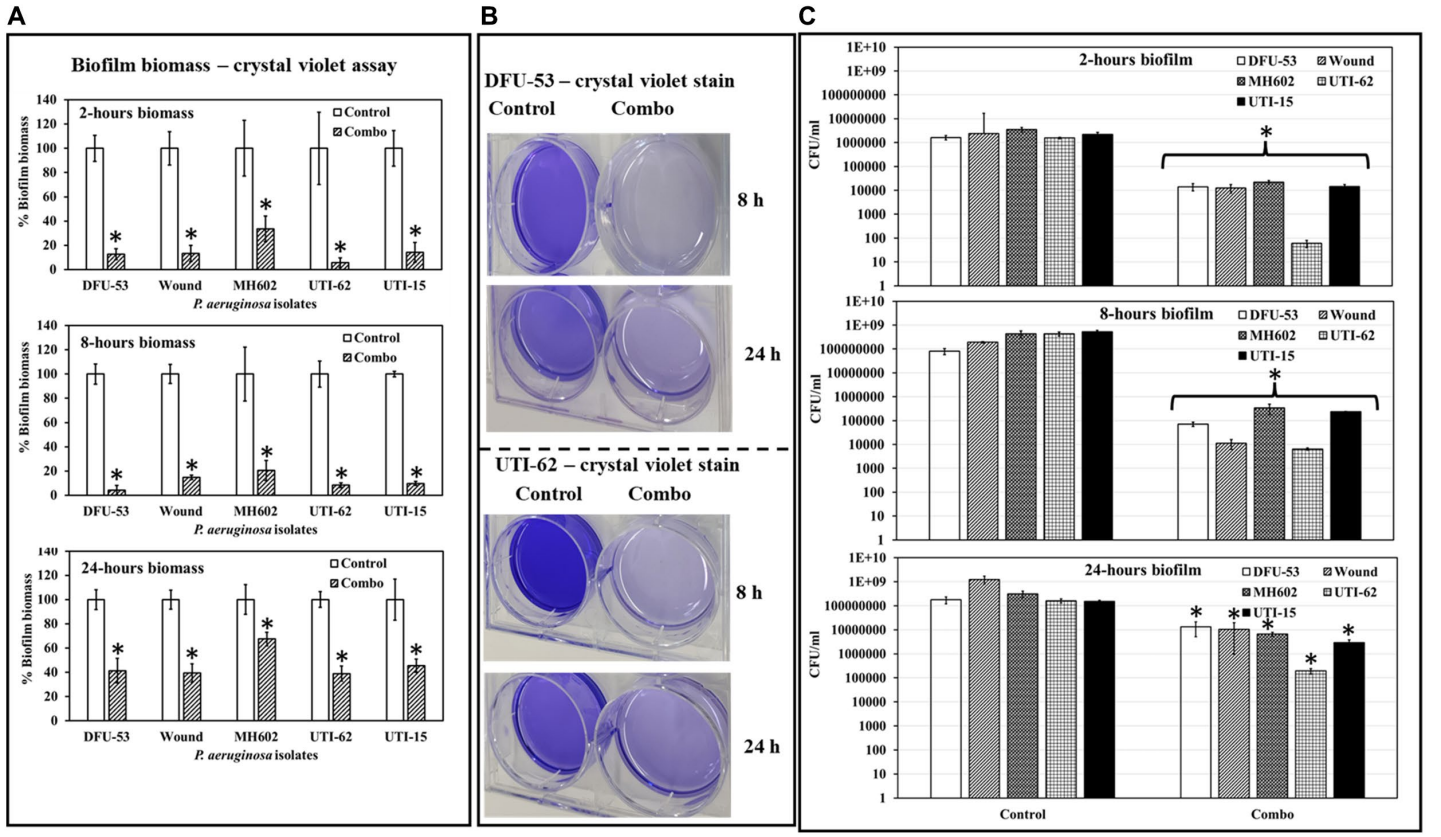
Quantification of *P. aeruginosa* biofilm biomass at 2, 8 and 24 h time points. **(A)** In the presence of a combo, the biomass is significantly less for all isolates **(A)**. **(B)** Photographic images showing control retain dense crystal violet stain compared to the biofilm grown in the presence of combo. **(C)** In the presence of a combo, the CFU/mL was significantly less at all time points than the control. ^*^Indicates the differences are statistically significant (*p* < 0.05) compared to the control. All experiments were conducted in *n* = 3 biological replicates.

At all-time points, *P. aeruginosa* grown in the presence of ascorbic acid-Fu-30 combo resulted in significantly less crystal violet staining (Figure 8A), suggesting the combination interferes with initial biofilm formation and maturation. Representative images of isolates DFU-53 and UTI-62 after 8 and 24 h incubation are given in Figure 8B. Similarly, there was a decrease in the number of colonies of all *P. aeruginosa* isolates after 2 (3–4 log_10_ reduction), 8 (3–4 log_10_ reduction) and 24 (1–3 log_10_ reduction) h incubation when grown in the presence of the combination (Figure 8C).

### Impact of tobramycin on pre-established biofilms grown in the presence of ascorbic acid and Fu-30

**Figure 9:**
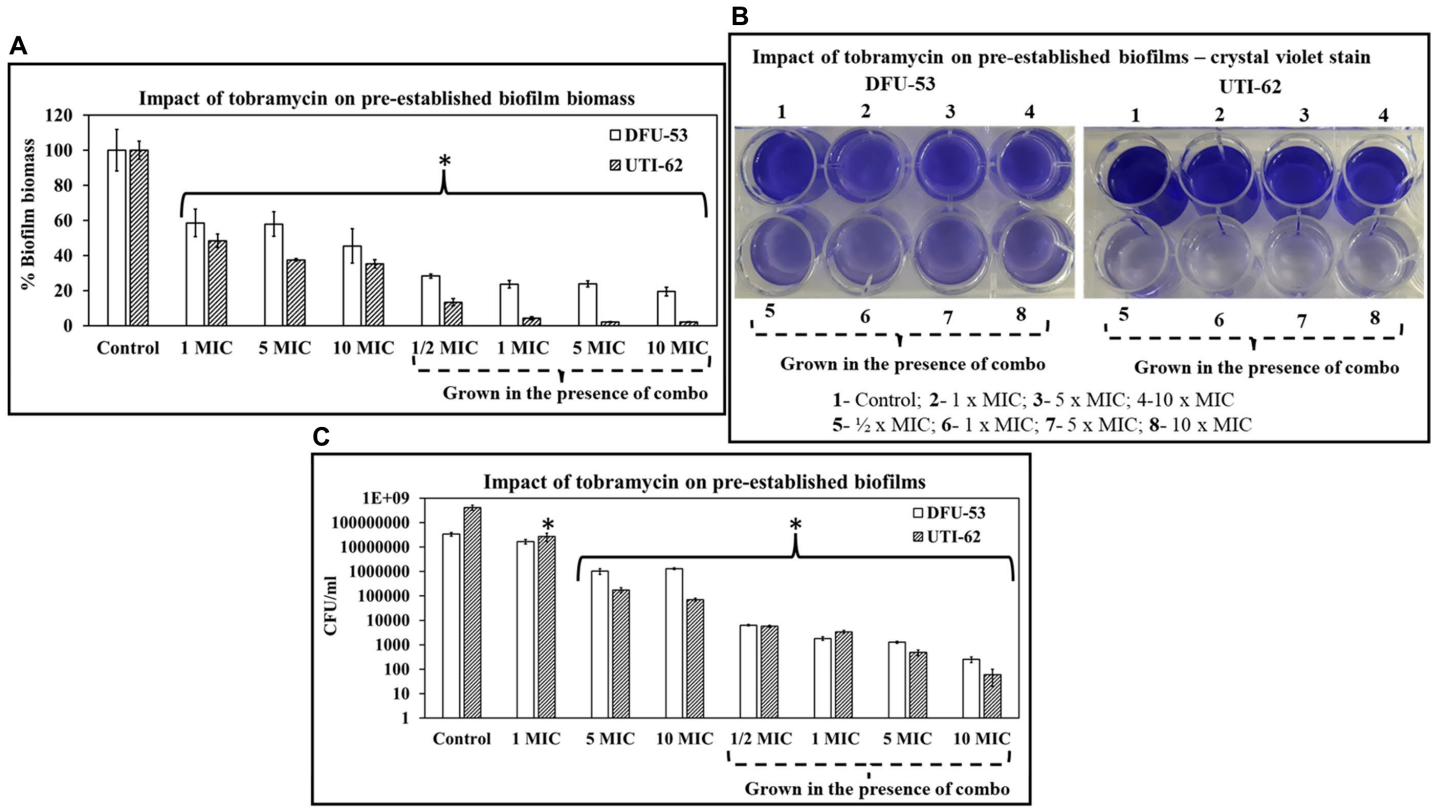
The impact of tobramycin and combo + tobramycin on *P. aeruginosa* pre-established biofilm. **(A–C)** On pre-established biofilms, combo + tobramycin treatment (4 h) exhibited a significant decrease in its biofilm biomass **(A)**, crystal violet stain retention **(B)** and CFU/mL in comparison to tobramycin alone treatment **(C)**. ^*^Indicates the differences are statistically significant (*p* < 0.05) compared to the control. All experiments were conducted in *n* = 3 biological replicates.

Figures 9A,B shows crystal violet data from biofilms grown in the combination of ascorbic acid and Fu-30 for 24 h plus tobramycin. The data suggests cells were more susceptible to tobramycin in the presence of ascorbic acid and Fu-30. Pre-established biofilm biomass was reduced 50%–60% and 40%–50% for DFU-53 and UTI-62, respectively, when treated with tobramycin alone (1–10 × MIC). In comparison, tobramycin treatment was more effective for biofilms grown in the presence of the combination, showing significantly reduced biomass for both DFU-53 (25%–32%) and UTI-62 (3%–17%).

DFU-53 and UTI-62 CFU/mL also exhibited a significant decrease when treated with tobramycin for the biofilm grown in the presence of ascorbic acid and Fu-30. When grown in the presence of the combination, the decrease in CFU/ml ranged between 3.5 log_10_ to 6.5 log_10_ for 1/2 MIC-10 MIC compared to the untreated control. Whereas when biofilms were grown without the combination treatment, tobramycin treatment exhibited a smaller decrease ranging from 0.2 log_10_ to 3.5 log_10_ (Figure 9C).

### The combination of ascorbic acid and Fu-30 was not cytotoxic to human fibroblast cell lines (HFF-1)

**Figure 10:**
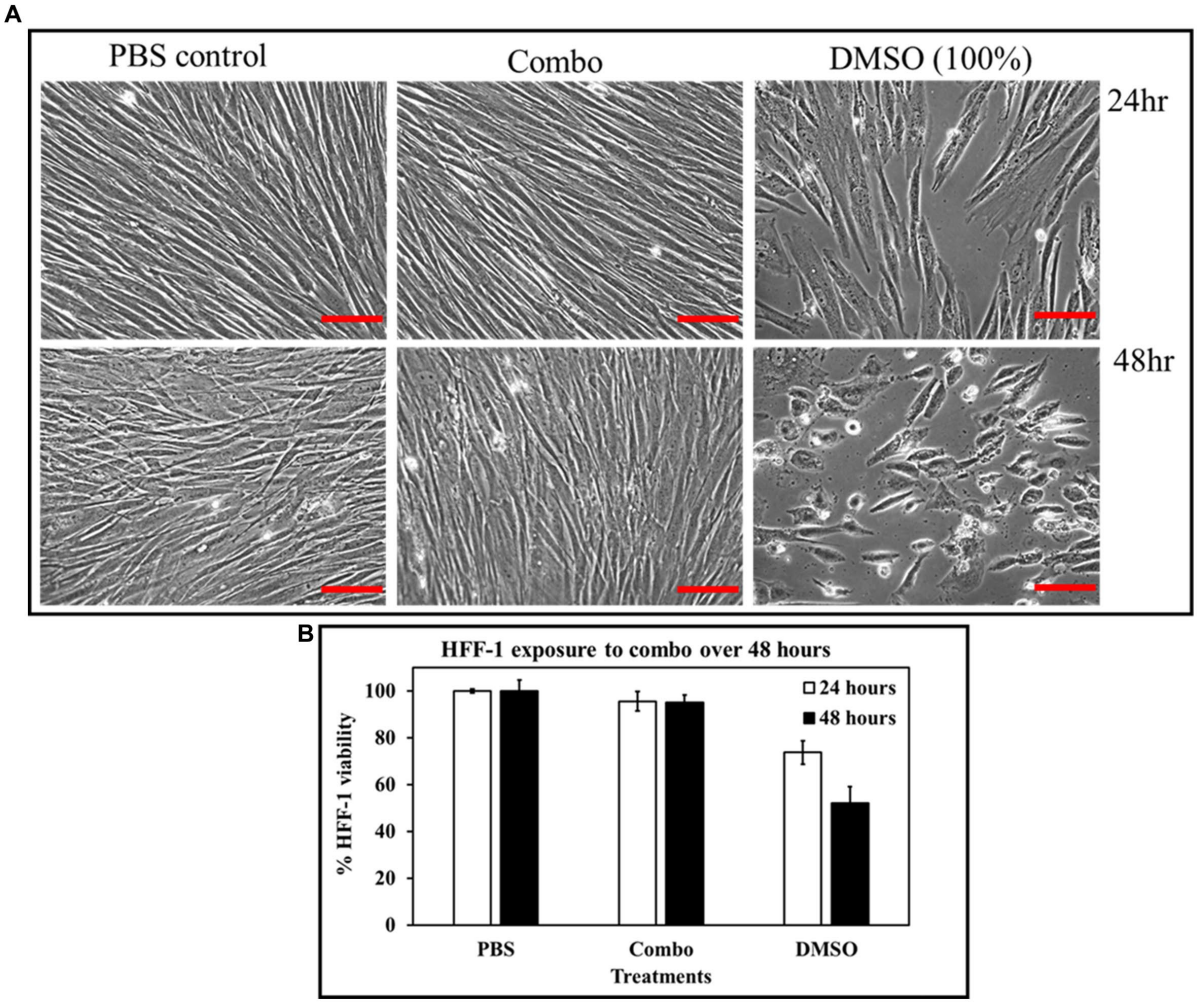
Cytotoxicity effect of the combo (AA 20 mM + Fu-30 20 μM) on HFF-1 cell lines. HFF-1, when exposed to combo, showed no cytotoxicity at both 24 and 48 h exposure time, whereas DMSO exhibited disruption in cell confluence and up to 50% cytotoxicity at 48 h time points **(A,B)**. Scale bar = 50 μm. All experiments were conducted in triplicates. ^*^Indicates *p* < 0.05.

Figures 10A,B demonstrates the impact of combination on human fibroblast cell lines. When HHF-1 cells were exposed to the combination of 20 mM ascorbic acid and 20 μM Fu-30, there was no evidence of cytotoxicity in microscopic images or when resazurin was used as an indicator of metabolic activity. Both the HFF-1 cell confluence and metabolic activity of combination treated cells looks similar to the PBS control (almost 100% survival cell viability). In contrast, the positive control of DMSO (100%) resulted in a 25% and 45% reduction in viability after exposure for 24 and 48 h, respectively.

## Discussion

Reduction of pyocyanin results in a change of the intrinsic pyocyanin blue colour to brown over time at acidic and physiological pH. Previous studies with the thiol antioxidant glutathione had shown that glutathione directly interacts with pyocyanin and results in a pyocyanin-glutathione conjugate product (Cheluvappa and Eri, 2015; Das et al., 2015). Ascorbic acid is a reducing agent and rapidly undergoes two consecutive one-electron donation processes to form the ascorbate radical and dehydroascorbic acid (Padayatty and Levine, 2016). At physiological pH, ascorbic acid is predominantly present as the ascorbate monoanion (by interacting with dissolved oxygen) (Buettner, 1988; Jiang et al., 2010; Back2health chiropractic wellness, 2020; Yin et al., 2022). A similar conjugate product to that with glutathione was predicted to have occurred between pyocyanin and ascorbic acid in the current study, given the evidence from the DFT model. Based on the DFT model, one of the very first probable interactions occurred through H-bonding between (i) the –CH_2_OH unit of ascorbic acid with –C=O of pyocyanin and (ii) the most acidic –OH unit of ascorbic acid with –C=O of pyocyanin (Figure 3; Supplementary Figure S4). Pyocyanin structure changed in the presence of ascorbic acid. The changes indicated that ascorbic acid perturbed the pyocyanin aromatic ring, not through the intrinsic acidity of ascorbic acid (as citric acid and dehydroascorbic acid had no effect), but most likely *via* the intrinsic antioxidant property of ascorbic acid. Furthermore, whilst pyocyanin can intercalate with DNA and stabilise *P. aeruginosa* biofilms (Das et al., 2015), the structural modulation of pyocyanin by ascorbic acid resulted in it losing its planar architecture, which ultimately may hinder pyocyanin’s ability to intercalate with nitrogenous bases of DNA (Figures 4B,C).

In *P. aeruginosa,* population-dependent QS is activated through the secretion of AHLs and binding of AHLs to regulatory receptors, initially LasR and RhlA to promote further biosynthesis of LasR, RhlA and PqsR receptors, which activate the complete QS system activating virulence factor production and biofilm formation (Lee and Zhang, 2015). The structure of ascorbic acid (five-membered lactone ring) is analogous to AHLs (Han et al., 2020), and Fu-30 is another analogue of AHLs and a known synthetic QS inhibitor (Zhao et al., 2018). Therefore, these two molecules may compete with *P. aeruginosa* intrinsic AHLs for LasR/RhlA interactions. The current study showed that Fu-30 was effective in reducing QS activity only during the initial hours of incubation, and after 24 h in the presence of Fu-30 *P. aeruginosa* could produce more GFP, that indicates activation of QS system and consequently impact pyocyanin biosynthesis (Figure 5; Supplementary Figure S6). On the other hand, ascorbic acid maintained its inhibition of the QS system and decreased pyocyanin production throughout the 24 h incubation, albeit at 1000-fold higher concentration. Dehydroascorbic acid also showed similar QS inhibition (tested for LasR and PqsR) to its reduced form (ascorbic acid) (Supplementary Figure S6D). This indicates a possible structural similarity between dehydroascorbic acid and AHLs, which leads to competition in binding to the AHL receptor(s) and inhibition of the QS system. The antioxidant property of ascorbic acid is needed for its independent interaction with pyocyanin.

The combination of ascorbic acid and Fu-30 was more effective (in comparison to Fu-30 and AA alone) in reducing GFP production for all three QS receptors at all time points. Reduction in QS activity consequently impaired pyocyanin production and hemolysis of rabbit blood cells (Figures 5, 6). Although ascorbic acid from 30 mM and above and Fu-30 at 30 μM, showed significant decreases in bacterial growth (in the case of ascorbic acid, probably due to the acidity of the growth environment, which was reduced to ≤pH 5 at 30 mM and further below to ≤pH 4 at 70 mM), it is important to note that the concentration of ascorbic acid (20 mM) and Fu-30 (20 μM) used in this study did not induce a significant reduction in *P. aeruginosa* growth (Supplementary Figure S6A). This indicates that the effects of the combination were not the result of any change in growth.

The combination of ascorbic acid and Fu-30 induced multiple changes, including to QS-mediated production of virulence factors such as hemolysin and pyocyanin, and ascorbic acid directly bound to pyocyanin resulting in its neutralisation of eDNA binding. Previous studies have shown that QS and pyocyanin are involved in biofilm development (Das et al., 2015, 2022), and pyocyanin binding to eDNA promotes *P. aeruginosa* surface adhesion, aggregation and biofilm formation (Das et al., 2015). In addition to the impact of the combination on biofilm formation, the current study showed that the combination rendered mature biofilms significantly more suspectable to tobramycin. This indicated that the combination might be a useful agent alone or combined with tobramycin to treat *P. aeruginosa* biofilm-mediated infections. Therefore, any possible toxicity of the combination was tested and shown to be non-cytotoxic to a human fibroblast cell line. This is an important requirement for developing any new antibacterial strategy. Fibroblasts are crucial in wound healing, and future research will use the combination-based strategy to treat infected wounds *in vivo* models.

To conclude, this study has, for the first time, shown that the antioxidant ascorbic acid can directly interact with pyocyanin and modulate its structure and its DNA binding ability. Additionally, in combination with the furanone, Fu-30 can significantly hinder the QS system of *P. aeruginosa* and inhibit virulence factor production, promote biofilm disruption and increase the sensitivity of biofilms to tobramycin. This indicates that the combination may be a new way to control the many infectious diseases caused by *P. aeruginosa*. Similar strategies can also be trailed by combining antioxidants with surfactins derived from other bacterial species or plant-based derivatives to facilitate inhibition of pathogenic bacteria virulence production and biofilm formation and its associated infections (Kimyon et al., 2016; Bhatwalkar et al., 2021; Ali et al., 2022).

## Data availability statement

The original contributions presented in the study are included in the article/Supplementary material, further inquiries can be directed to the corresponding author.

## Ethics statement

All clinical isolates used in this study were obtained from Royal Prince Alfred Hospital (Sydney, Australia) and the Liverpool Hospital (Sydney, Australia). The Tissue Act (NSW, 1983) did not require the study to be reviewed or approved because: all isolates were de-identified by the hospitals concerned prior to being gifted to us, all species/isolates were from their historical culture collections, and they were not collected from patients as part of this study. Therefore, this study does not warrant a requirement to obtain ethics approval.

## Author contributions

TD designed, conducted the experiments, and wrote the manuscripts. BD and SS conducted the experiments and wrote the manuscript. BY conducted the experiments. BA, MW, MM, and NK edited the manuscript. All authors contributed to the article and approved the submitted version.

## Funding

This work was supported by the KKU Deanship of Scientific Research to BA (grant number: RGP.1/332/43).

## Conflict of interest

The authors declare that the research was conducted in the absence of any commercial or financial relationships that could be construed as a potential conflict of interest.

## Publisher’s note

All claims expressed in this article are solely those of the authors and do not necessarily represent those of their affiliated organizations, or those of the publisher, the editors and the reviewers. Any product that may be evaluated in this article, or claim that may be made by its manufacturer, is not guaranteed or endorsed by the publisher.

## Footnotes

^1^https://www.graphpad.com/quickcalcs/ttest1.cfm
