# Supplementary material for: Ascorbic acid modulates the structure of the Pseudomonas aeruginosa virulence factor pyocyanin and ascorbic acid-furanone-30 combination facilitate biofilm disruption
Source: Front Microbiol. 2023 Jul 13;14:1166607. doi: 10.3389/fmicb.2023.1166607 (PMC10381918; doi:10.3389/fmicb.2023.1166607)
Supplement: Supplementary file 1 [file Data_Sheet_1.PDF]

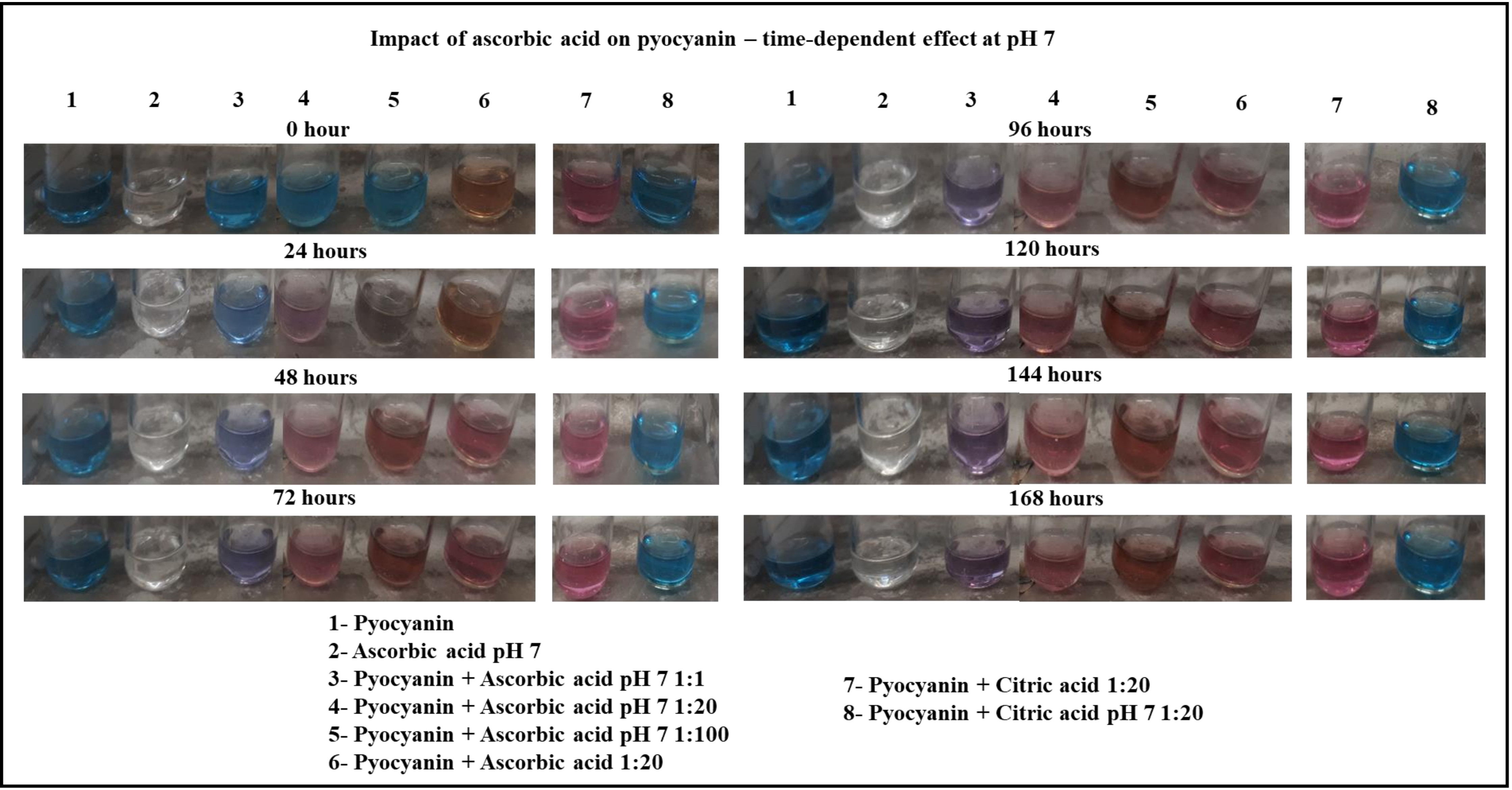

Supplementary Figure 1. Showing change in pyocyanin colour as the impact of the ascorbic acid (at intrinsic acidic pH) and neutralised pH. The colour change indicates that pyocyanin and ascorbic acid interaction is concentration-dependent and time-dependent. Additional control using acidic and neutralised citric acid showed no change in pyocyanin colour.

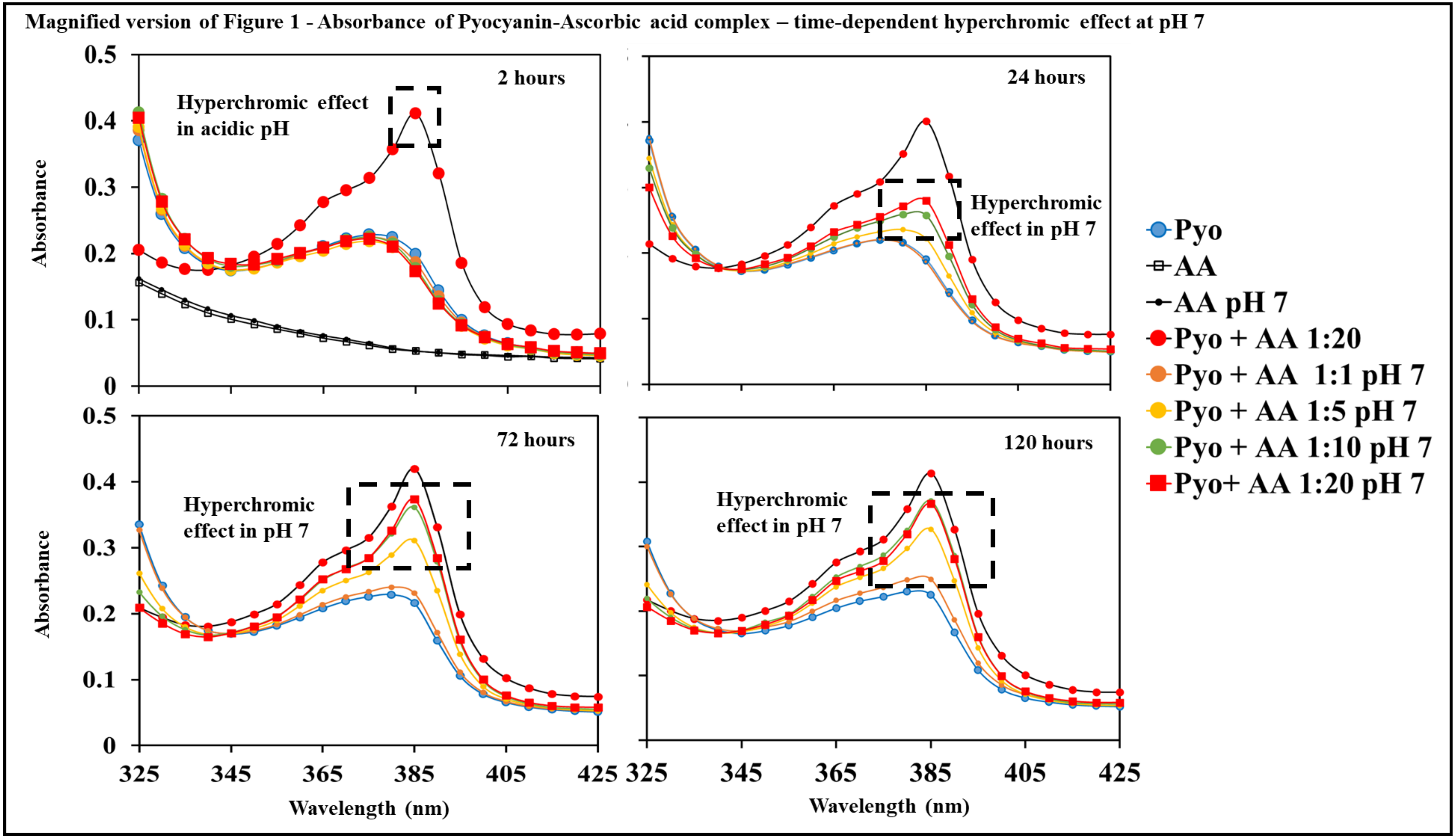

Supplementary Figure 2. Magnified version of main figure 1 showing the spectrophotometer analysis of ascorbic acid (AA) impact on pyocyanin (Pyo) absorbance at region between 325-425 nm. Ascorbic acid (acidic pH) showed immediate hyperchromic (seen in 2 h graph - top left), whereas in neutral pH, the hyperchromic shift was gradual dependent on concentration of ascorbic acid and was more prominent at later hours. The hyperchromic effect in the graph is indicated with square dotted box.

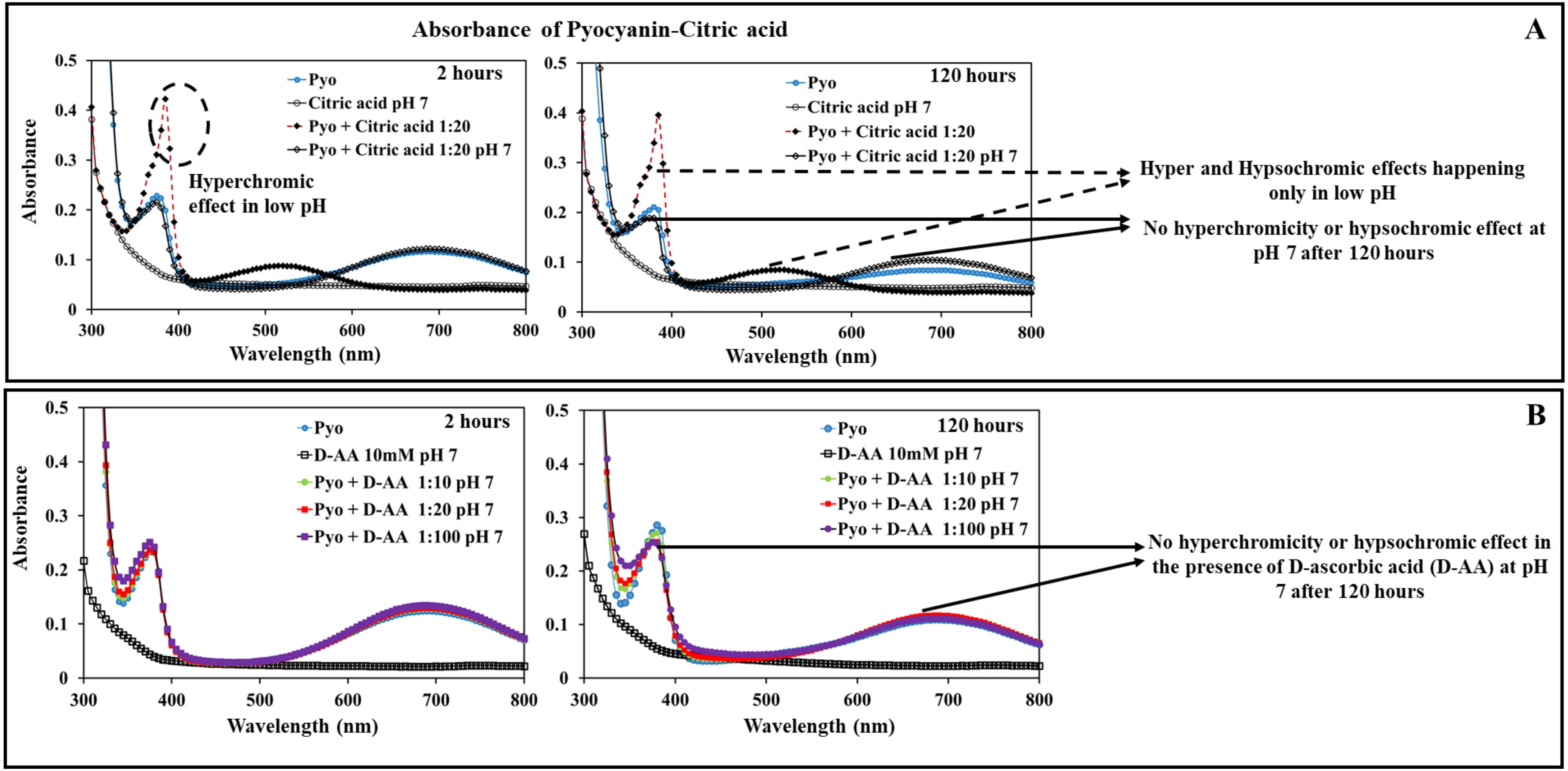

Absorbance of Pyocyanin-Citric acid
A
2 hours
Pyo
Citric acid pH 7
Pyo + Citric acid 1:20
Pyo + Citric acid 1:20 pH 7
Hyperchromic effect in low pH
Absorbance
Wavelength (nm)
120 hours
Hyper and Hypsochromic effects happening only in low pH
No hyperchromicity or hypsochromic effect at pH 7 after 120 hours
B
D-AA 10mM pH 7
Pyo + D-AA 1:10 pH 7
Pyo + D-AA 1:20 pH 7
Pyo + D-AA 1:100 pH 7
No hyperchromicity or hypsochromic effect in the presence of D-ascorbic acid (D-AA) at pH 7 after 120 hours

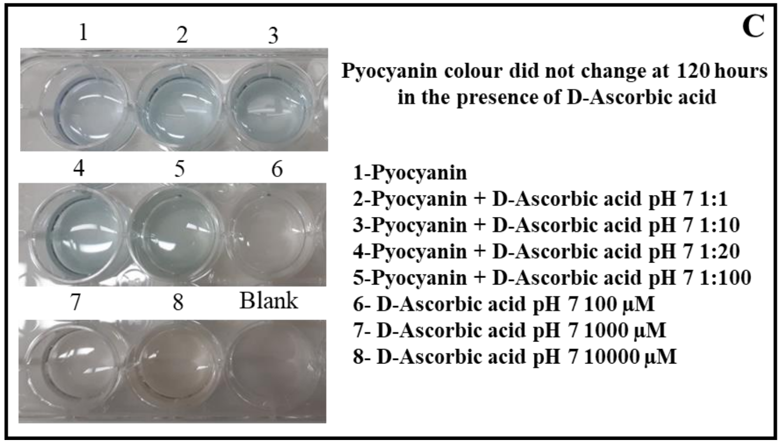

Supplementary Figure 3. Spectrophotometer analysis of the impact of citric acid (A) and Dehydroascorbic acid/oxidised ascorbic acid (D-AA) (B) on pyocyanin (Pyo) absorbance. The citric acid (acidic pH) showed immediate_hyperchromic and hypsochromic shifts at 385 nm and 695 nm, respectively; however, under neutralised buffer conditions, citric acid has no impact on pyocyanin absorbance peaks. Similarly, D-AA does not affect the pyocyanin absorbance peak, even at a 1:100 ratio to pyocyanin even after 120 h. Also, D-AA did not change pyocyanin colour even after 120 h (C). Indicates that ascorbic acid intrinsic antioxidant property is essential for pyocyanin modulation.

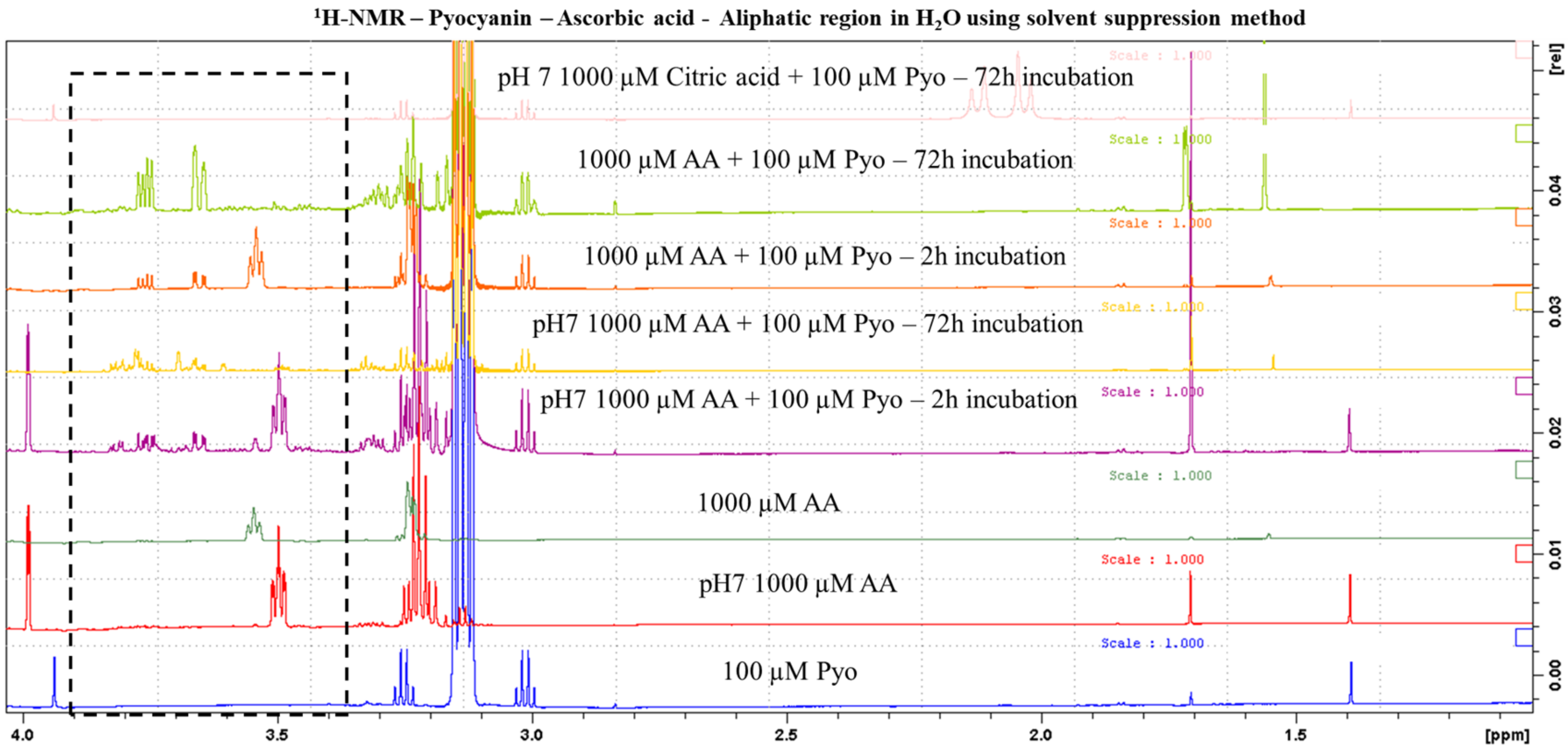

Supplementary Figure 4. ^{1}H-NMR analysis of the impact of Ascorbic acid (AA) on pyocyanin in the aliphatic region. The aliphatic part of pyocyanin showed mild modulation in its_characteristic NMR signals upon forming a complex with ascorbic acid at both acidic and neutralised pH. At the same time, the aliphatic region of ascorbic acid showed multiple additional new peaks in the range between 3.5 to 4.0 ppm at both acidic and neutral pH. This indicates modulation in ascorbic acid structure due to complex formation between pyocyanin-ascorbic acid adducts. The aliphatic region of citric acid (pH 7) encountered no changes or construction of new peaks and retained original pyocyanin peaks at 3 and 3.25 ppm.

Supplementary Figure 5. DFT calculations. The primary energy-minimised structures of Vitamin C and Pyocyanin were obtained using the MM2 function implemented in Chem3D (Suppl. ref 1). For DFT optimisation of the geometries, B3LYP and standalone functional B97D3 (as implemented in the Gaussian09 package) were tested (Suppl. ref 2-4). In all the cases, a 6-31 G (d,p) basis set was used for all the atoms (C, H, N and O).

Cartesian coordinates for (a)

0 (charge) 1 (spin multiplicity)

C	5.00643500	-0.48976100	-1.85068300
C	4.39007900	0.51401600	-2.63102400
C	3.29028900	1.17417600	-2.13357100
C	2.77900500	0.85937600	-0.84928800
C	3.42538600	-0.13887100	-0.05425000
C	4.54404500	-0.81724500	-0.58983200
N	1.67735200	1.52458000	-0.41569400
C	1.15714300	1.21160900	0.74871500
C	1.74540700	0.20559700	1.62812400
N	2.91916400	-0.38886600	1.20281300
C	-0.09751700	1.91783100	1.15276000
C	-0.64725500	1.55048500	2.40923500
C	-0.04543400	0.58100900	3.19435800
C	1.13255300	-0.10106400	2.83717700
O	-0.61617200	2.77303600	0.37351600
C	3.59910900	-1.31549300	2.12096300
H	5.03371500	-1.60876700	-0.03933900
H	5.86216700	-1.02621100	-2.24863600
H	4.77873500	0.75083100	-3.61560900
H	2.77699300	1.94231800	-2.70216300
H	1.52373300	-0.86100800	3.49818400
H	-0.50726900	0.32057300	4.14340800
H	-1.55704900	2.04458500	2.73467100
H	3.11215400	-2.29411500	2.10748600
H	3.55906500	-0.90273800	3.12833500
H	4.64285700	-1.41867900	1.84243800
C	-2.60383700	-0.23934100	-0.30782600
C	-3.02624300	-1.65842700	-0.04622100
C	-1.98522700	-2.48033500	-0.30162600

C	-0.88115100	-1.67228900	-0.78643500
O	-1.26752600	-0.35619200	-0.83796500
C	-3.53929300	0.51202900	-1.26696000
C	-3.11781800	1.95951200	-1.51880500
O	-3.22145300	2.72856600	-0.32561000
O	0.23941500	-2.02860900	-1.12425200
O	-1.91742400	-3.84006800	-0.17628700
O	-4.25999300	-1.96945500	0.38288200
O	-4.84537200	0.48384200	-0.66480600
H	-3.60639000	-0.03096900	-2.21461200
H	-2.10287500	1.98691800	-1.93068000
H	-3.80138400	2.38554000	-2.26217800
H	-2.54196000	0.32279600	0.63181200
H	-2.32164800	2.75525300	0.08187300
H	-1.02776500	-4.11381700	-0.45528200
H	-4.80860300	-1.16339100	0.24103200
H	-4.83594100	1.19503300	0.00073800

Cartesian coordinates for (b)

0 (charge) 1 (spin multiplicity)

C	1.71687800	4.05906000	0.41714300
C	0.61633200	3.43311700	1.04490500
C	0.58165800	2.06096800	1.13153800
C	1.63510700	1.27944900	0.59206900
C	2.73783500	1.91812200	-0.05991000
C	2.75886200	3.33063100	-0.12507600
N	1.55720000	-0.06974900	0.71469800
C	2.52967600	-0.81814600	0.25053000
C	3.70347400	-0.25716600	-0.40975700
N	3.72801400	1.11529900	-0.58500900
C	2.40267500	-2.29411200	0.43028200
C	3.49175400	-3.07639400	-0.03384200
C	4.59170800	-2.48345000	-0.62987300
C	4.72545800	-1.09674600	-0.83374700
O	1.37571400	-2.79829600	0.97932400

C	4.85666200	1.68407900	-1.33788800
H	3.58678000	3.85997400	-0.57552200
H	1.75428800	5.14248900	0.36082400
H	-0.18612500	4.03444500	1.45831500
H	-0.23624300	1.53538400	1.61334100
H	5.62254200	-0.71376800	-1.29834100
H	5.40682600	-3.11997700	-0.96467500
H	3.43725500	-4.15261700	0.09236600
H	5.02240400	1.08162800	-2.23118900
H	5.76080300	1.68049900	-0.72368800
H	4.63135600	2.69721500	-1.65152400
C	-3.33757100	-0.02660100	-0.31224100
C	-2.72402700	-0.36819700	1.01425000
C	-1.68836900	-1.21929800	0.83579700
C	-1.59612300	-1.50181300	-0.59179300
O	-2.58150300	-0.80179700	-1.26260700
C	-4.83963400	-0.35921700	-0.38296500
C	-5.48139700	0.02409500	-1.70925100
O	-5.44008700	1.45149100	-1.79890700
O	-0.81961000	-2.22252400	-1.19991300
O	-0.89867200	-1.77867300	1.78542800
O	-3.19196300	0.13615600	2.17245800
O	-5.48331200	0.34811200	0.68847400
H	-4.97411500	-1.42967000	-0.20188000
H	-4.94111800	-0.44841300	-2.53952600
H	-6.51581300	-0.33671800	-1.71730500
H	-3.19452400	1.03429800	-0.54876700
H	-6.18289700	1.73848600	-2.34663600
H	-0.00916500	-2.04358400	1.39781400
H	-4.09427600	0.47523700	1.97734300
H	-5.65476700	1.24020800	0.33613700

Cartesian coordinates for (c)

0 (charge) 1 (spin multiplicity)

C	4.44204100	-0.34529200	-0.57137700

C	4.02200000	-0.42747500	-1.92149800
C	2.79097200	0.08876600	-2.28116000
C	1.95334300	0.70625000	-1.31322400
C	2.40864800	0.82592800	0.04774200
C	3.66123200	0.26623300	0.40087600
N	0.71532300	1.11601000	-1.70636200
C	-0.09985600	1.63582800	-0.80418400
C	0.29930700	1.86211200	0.58391700
N	1.58519300	1.47646000	0.94455800
C	-1.51413600	1.91033800	-1.22916800
C	-2.35527000	2.51915800	-0.25236700
C	-1.90335100	2.74548700	1.04261400
C	-0.60357000	2.42125900	1.48703800
O	-1.92505900	1.52160700	-2.37264500
C	1.97801000	1.66342000	2.35080100
H	4.01772500	0.28446400	1.42464900
H	5.39806800	-0.77698400	-0.27877400
H	4.65733800	-0.91091600	-2.66093300
H	2.41342600	0.02017200	-3.29951800
H	-0.34655100	2.58145800	2.52745300
H	-2.59311000	3.17208300	1.77181700
H	-3.38397100	2.74092700	-0.53112700
H	1.41998100	0.96280800	2.98170200
H	1.75002600	2.69126400	2.64655800
H	3.04610200	1.50308200	2.47162600
C	-1.36959900	-1.22077100	0.09927800
C	-0.66117700	-1.22930500	1.42491100
C	0.58948500	-1.73203200	1.24546400
C	0.71106700	-2.17371600	-0.13390500
O	-0.47598100	-1.90645800	-0.81062800
C	-2.78463300	-1.81579300	0.10431600
C	-3.50591300	-1.63620600	-1.24321800
O	-3.92943500	-0.27875400	-1.41552700
O	1.66490400	-2.70927900	-0.69430700
O	1.59710000	-1.85707400	2.16529300

O	-1.23055200	-0.74622300	2.54381400
O	-3.51911300	-1.10945500	1.12899500
H	-2.74668500	-2.87514800	0.38701200
H	-2.85898200	-1.97509200	-2.06494300
H	-4.41430800	-2.25290400	-1.23580900
H	-1.45747000	-0.18675100	-0.24677200
H	-3.19788100	0.25000400	-1.80921200
H	2.39066900	-2.14757500	1.67859000
H	-2.19074300	-0.63677400	2.31495500
H	-3.81378100	-0.29146500	0.67425500

Cartesian coordinates for (d)

0 (charge) 1 (spin multiplicity)

C	-2.14561400	2.62172600	1.56296400
C	-1.76990700	1.88144900	2.71063100
C	-0.56981000	1.19221500	2.71761700
C	0.27884400	1.21013800	1.57941300
C	-0.11561000	1.95854200	0.41712300
C	-1.34082900	2.66817400	0.43077500
N	1.40180600	0.43647500	1.59197600
C	2.18915000	0.44945800	0.53124200
C	1.95034400	1.30298100	-0.62855600
N	0.71548500	1.93689800	-0.68380400
C	3.35321700	-0.48987000	0.53118700
C	4.34643400	-0.24988500	-0.45838900
C	4.11821200	0.66800400	-1.48043400
C	2.92960300	1.42070400	-1.61168900
O	3.39950700	-1.44366800	1.38028600
C	0.23485900	2.48996700	-1.96295600
H	-1.65063100	3.25792700	-0.42582000
H	-3.08413000	3.17394900	1.56312700
H	-2.42517900	1.85671000	3.57908000
H	-0.25153500	0.60118900	3.57396100
H	2.81237900	2.09432600	-2.45442300
H	4.88160500	0.79466000	-2.24894000
H	5.24798200	-0.86032100	-0.44643800

H	0.78554400	2.01912300	-2.77770600
H	0.37470400	3.57632900	-1.99697300
H	-0.82396200	2.24285500	-2.07583000
C	-1.58779300	-0.97482300	-0.50565000
C	-1.08532800	-1.70048700	0.70842000
C	0.19289700	-2.11613000	0.48909700
C	0.53245500	-1.79495800	-0.89434000
O	-0.53794100	-1.12496900	-1.49042400
C	-2.94915200	-1.46439200	-1.02723400
C	-3.43644100	-0.65364900	-2.22187400
O	-3.56004900	0.71203500	-1.78737700
O	1.55871100	-2.01734800	-1.53177500
O	1.03588200	-2.76304700	1.35329100
O	-1.83467300	-1.83122700	1.81800300
O	-3.87999100	-1.34835000	0.07398100
H	-2.88829600	-2.52695100	-1.29128400
H	-2.71884000	-0.75045900	-3.05209800
H	-4.40802200	-1.05185700	-2.54819900
H	-1.68181100	0.09496600	-0.27712200
H	-4.21940400	1.13721100	-2.35624900
H	1.90146300	-2.25327400	1.39271000
H	-2.75650700	-1.60032400	1.53526600
H	-4.12116700	-0.40206600	0.09776600

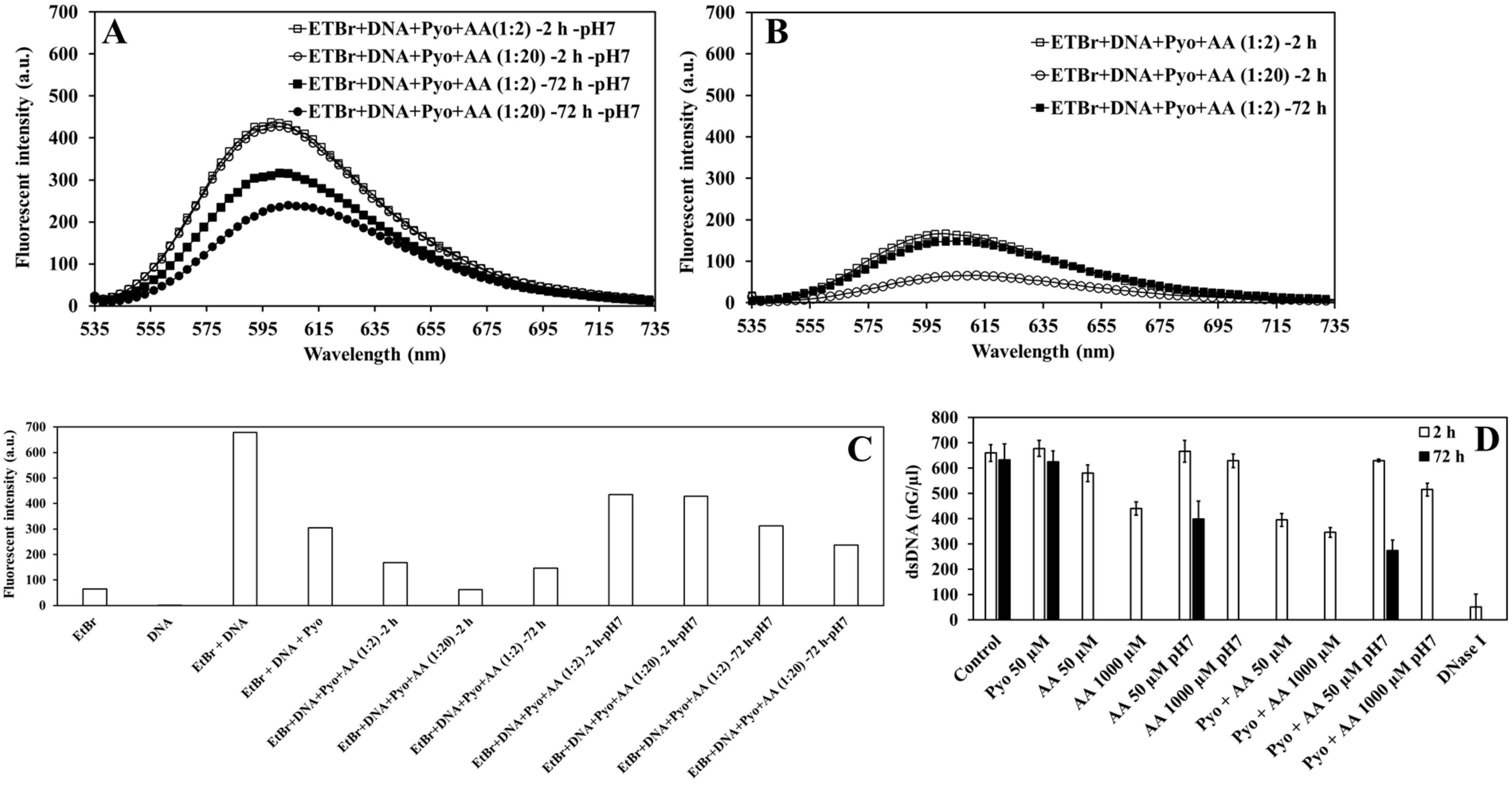

Supplementary Figure 6. Fluorescent spectroscopy analysis showing EtBr displacement analysis. Ascorbic acid inhibits pyocyanin binding to DNA (A-C). Qubit fluorometry data demonstrates that ascorbic acid (AA) in its intrinsic pH cleaves dsDNA faster than its neutral pH version. * indicates the differences are statistically significant ($p < 0.05$) compared to the control. All experiments were conducted in triplicates.

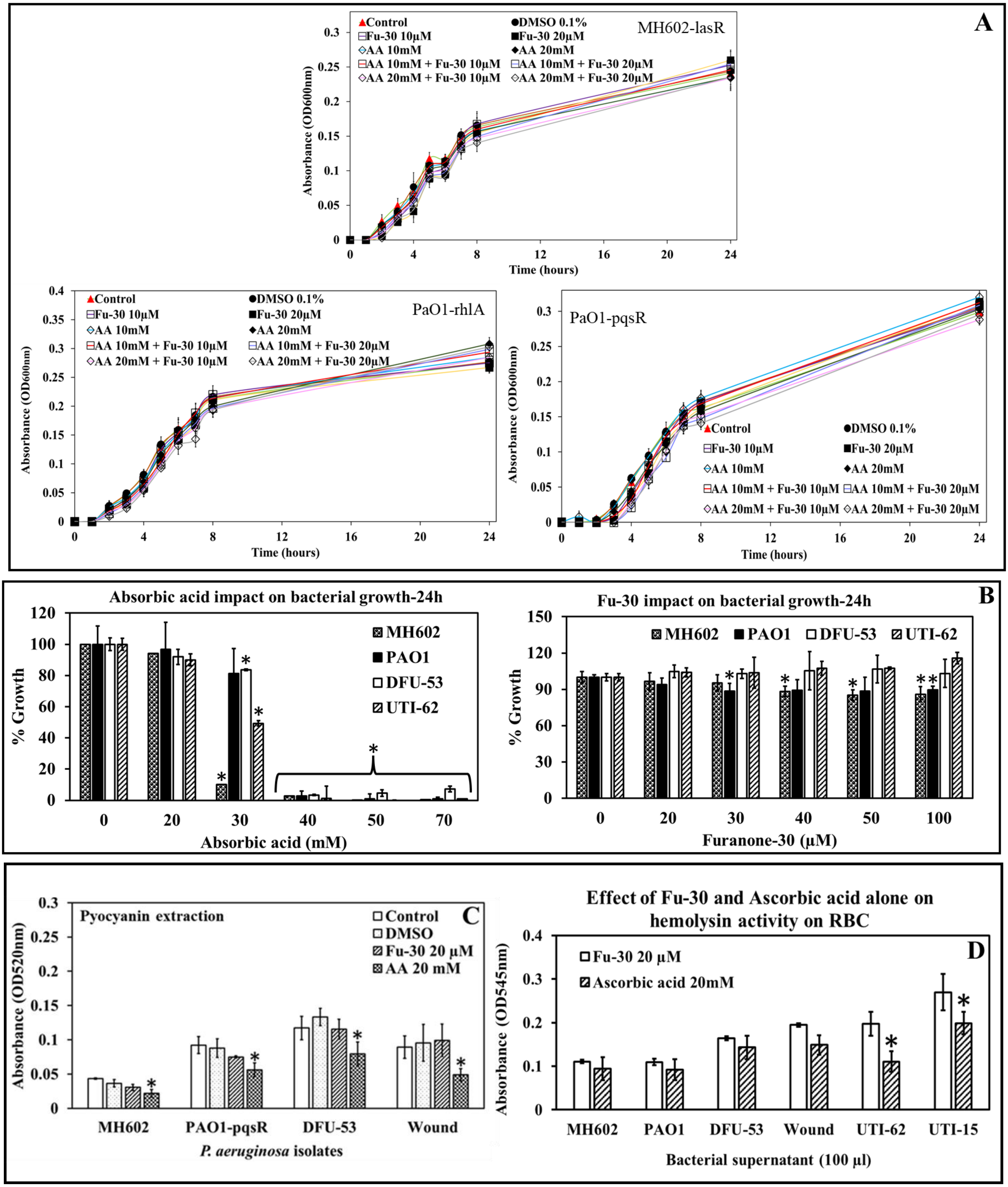

Supplementary Figure 7. Impact of Ascorbic acid (AA) and Furanone-30 (Fu-30) and combination of both on *P. aeruginosa* growth. At concentrations of AA (20 mM) and Fu-30 (20 µM), the differences in growth are

not significant (A). The AA concentration from 30 mM and Fu-30 from 30 µM showed significant differences in overall growth; the differences are larger with AA (B). Impact of individual compounds Fu-30 and ascorbic acid on pyocyanin production (C). Impact of individual compounds Fu-30 and ascorbic acid on hemolysin activity on rabbit blood cells (D). All experiments were conducted in triplicates. * indicates $p < 0.05$.

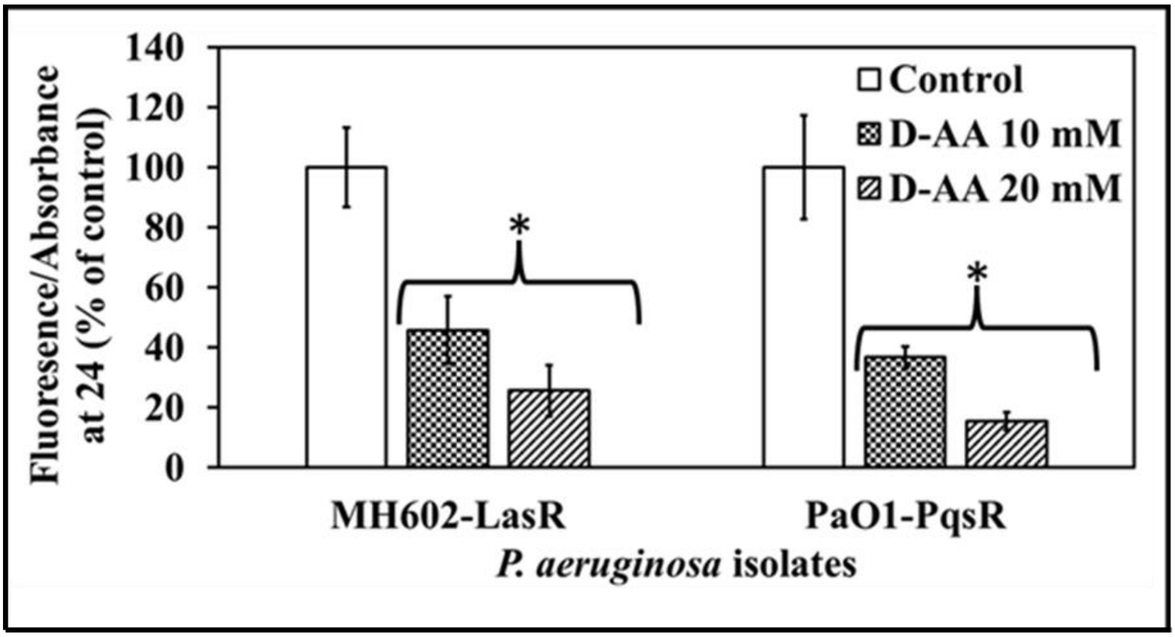

Supplementary Figure 8. Impact of Dehydroascorbic acid (D-AA) on GFP/absorbance. Decrease in fluorescence/absorbance indicates D-AA mediated inhibition of QS (LasR and PqsR) receptors at 24 h. All experiments were conducted in triplicates. * indicates $p < 0.05$.

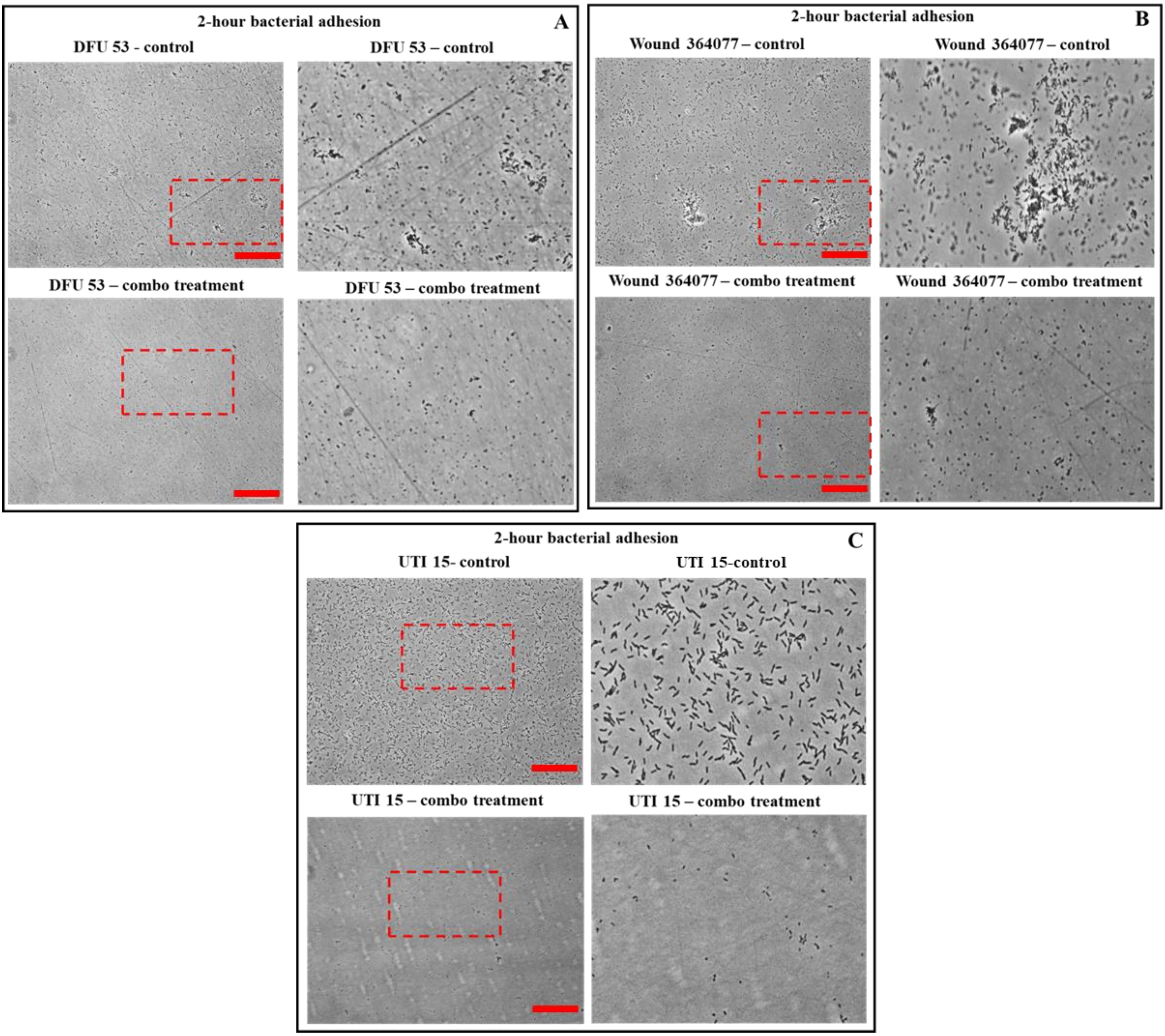

Supplementary Figure 9. The microscopic images represent the adhesion of *P. aeruginosa* clinical isolates at 2 h time points (A-C). In the presence of a combo, the bacterial adhesion drastically reduced. Scale bar = 50µm.

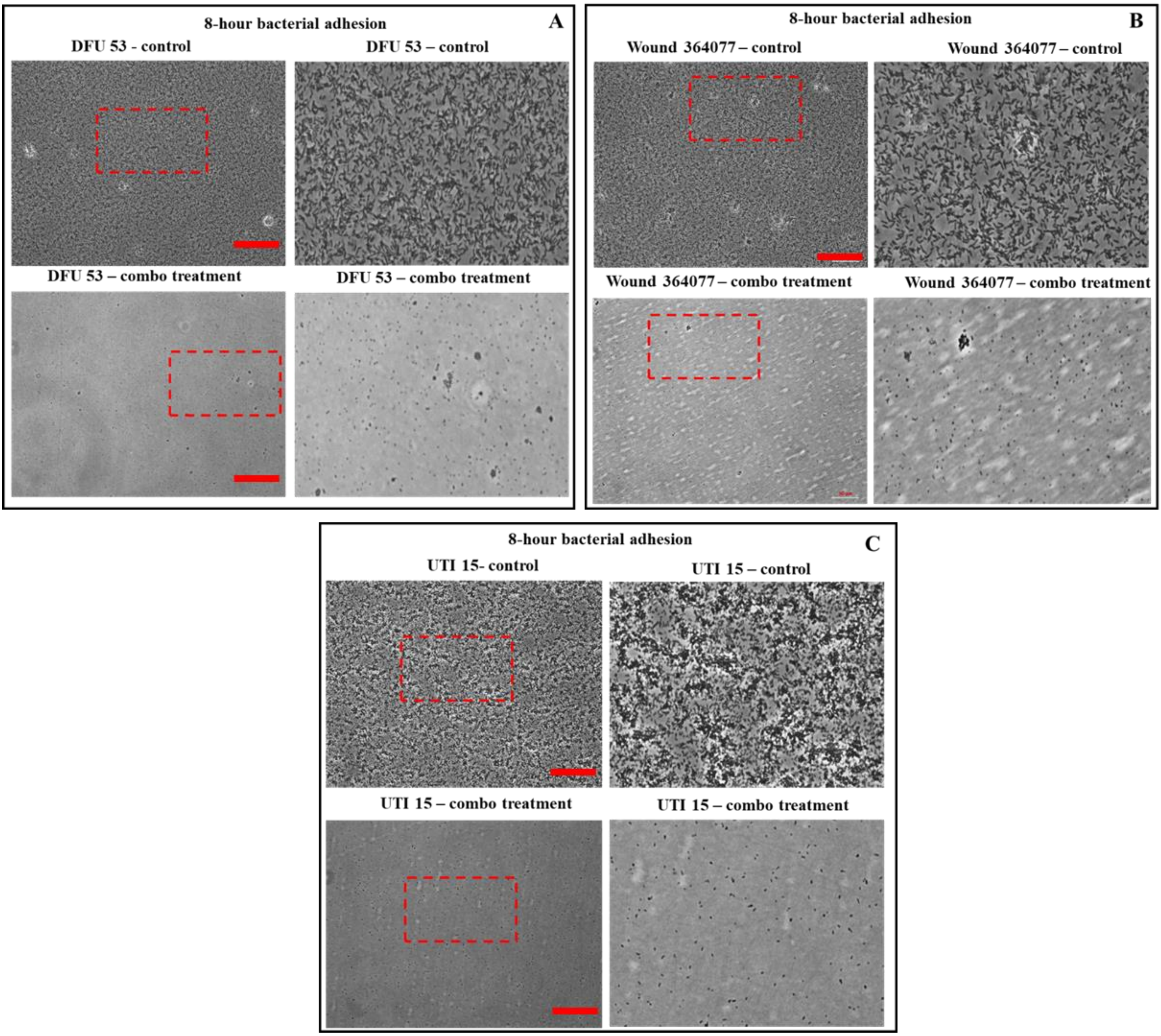

Supplementary Figure 10. The microscopic images represent the adhesion of *P. aeruginosa* clinical isolates at 8 h time points (A-C). In the presence of a combo, the bacterial adhesion drastically reduced. Scale bar = 50µm.

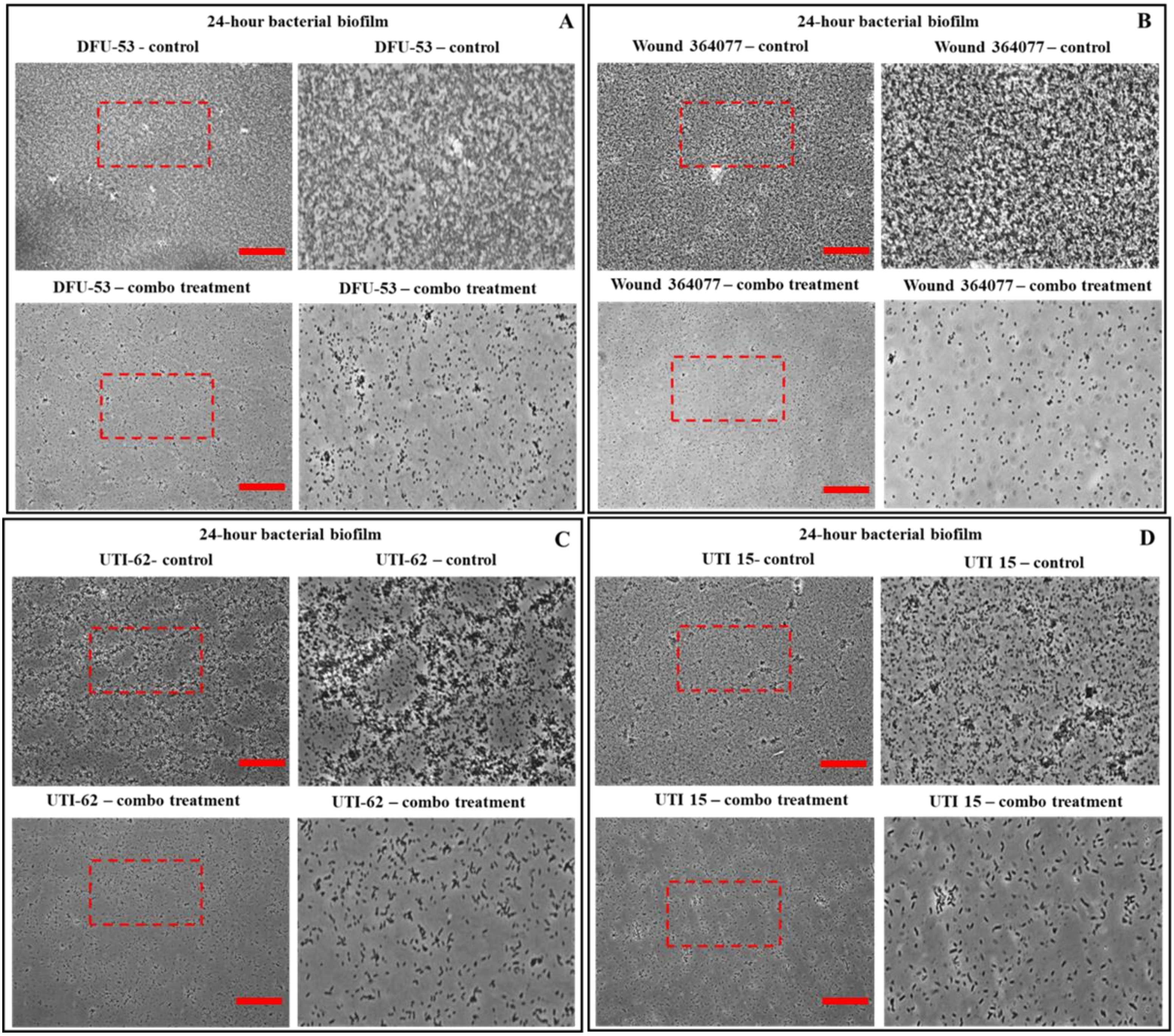

Supplementary Figure 11. The microscopic images represent the biofilms of *P. aeruginosa* clinical isolates at 24 h time points (A-D). In the presence of a combo, the biofilm formation ability of isolates is drastically hindered. Scale bar = 50µm.

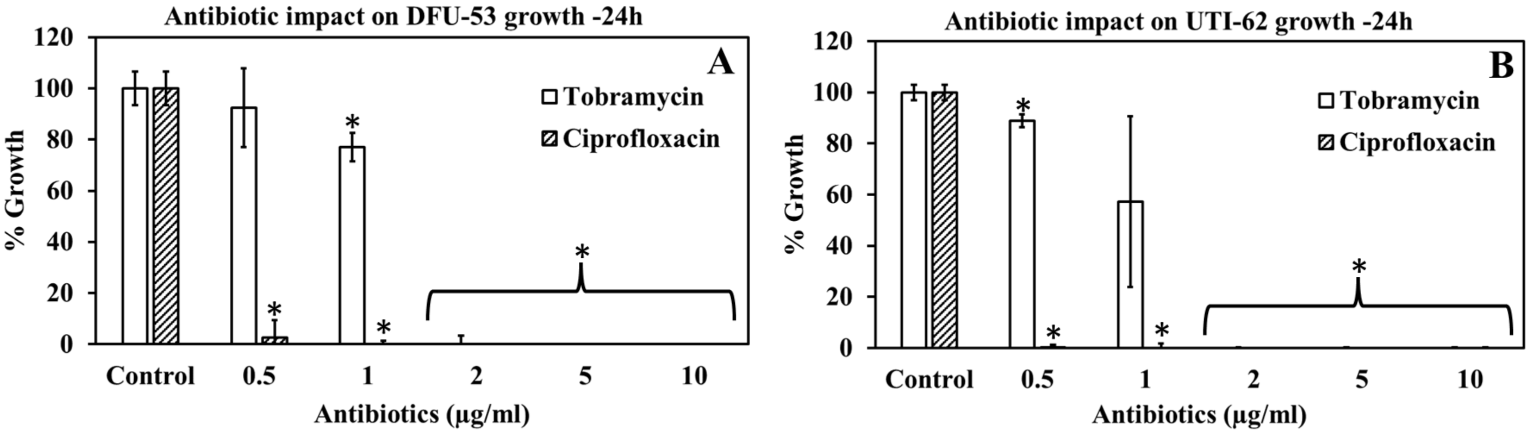

Supplementary Figure 12: MIC of tobramycin and ciprofloxacin on *P. aeruginosa* clinical isolates DFU-53 and UTI-62 (A and B). For tobramycin and ciprofloxacin, the MICs are 2 µg/ml and 0.5 µg/ml, respectively, for both clinical isolates. All experiments were conducted in triplicates. * indicates $p < 0.05$.
